# Graph Based, Adaptive, Multiarm, Multiple Endpoint, Two‐Stage Designs

**DOI:** 10.1002/sim.70237

**Published:** 2025-12-02

**Authors:** Cyrus Mehta, Ajoy Mukhopadhyay, Martin Posch

**Affiliations:** ^1^ Cytel Corporation Cambridge MA USA; ^2^ Harvard T.H. Chan School of Public Health Boston MA USA; ^3^ Medical University of Vienna Austria

**Keywords:** adaptive design, combining p values, conditional error rate, graphs, group sequential, hierarchical testing, MAMS design, multiple endpoints

## Abstract

The graph‐based approach to multiple testing is an intuitive method that enables a study team to represent clearly, through a directed graph, its priorities for hierarchical testing of multiple hypotheses, and for propagating the available type‐1 error from rejected or dropped hypotheses to hypotheses yet to be tested. Although originally developed for single‐stage nonadaptive designs, we show how it may be extended to two‐stage designs that permit early identification of efficacious treatments, adaptive sample size re‐estimation, dropping of hypotheses, and changes in the hierarchical testing strategy at the end of stage one. Two approaches are available for preserving the familywise error rate in the presence of these adaptive changes, the p value combination method, and the conditional error rate method. In this investigation, we will present the statistical methodology underlying each approach and will compare the operating characteristics of the two methods in a large simulation experiment.

## 1 Introduction

A common design issue in a confirmatory clinical trial comparing one or more treatment arms to a common control arm is how to perform hypothesis tests for more than one efficacy endpoint while preserving strong control of the familywise error rate (FWER). For example in a double‐blind clinical trial of patients with acute exacerbation of schizophrenia (ClinicalTrials.gov identifier NCT01469039), two doses of the experimental drug ALK‐9072 (350 mg/day or 500 mg/day) were compared to a matching control drug. The primary endpoint was the change from baseline in the PANSS total score at week 12. A key secondary endpoint was the change from baseline in the PANSS negative symptoms score. The trial would be considered a success if efficacy could be demonstrated with either dose for the primary endpoint. Once efficacy is demonstrated for the primary endpoint, there is interest in also filing a claim for the secondary endpoint. In settings such as this, a convenient way to conceptualize and display the study team's strategy for testing the various hypotheses in a hierarchical manner, while recognizing that some hypotheses are more important than others, is through a graph with weighted nodes representing the various hypotheses to be tested, and directed edges for propagating the weights from rejected hypotheses to ones yet to be tested.

Graph‐based approaches were developed in [1, 2] and [3] to construct flexible sequentially rejective Bonferroni‐based tests in single‐stage designs and several software implementations exist [4, 5, 6]. The graphs are a convenient way to define weights in a closed testing procedure, [7], where each intersection hypothesis is tested by a weighted Bonferroni test. Subsequently, [8] showed how, by separating the weighting strategy from the testing strategy, more efficient weighted Simes and Dunnett type tests could replace Bonferroni tests when additional information about the correlation structure among the hypotheses was available. Xi et al. [9] extended the procedure to weighted mixed tests for settings where the correlation between test statistics is only known for subsets of test statistics. Next, these testing procedures were extended to group sequential tests incorporating interim analyses. First, [10, 11] considered the case where the correlation between test statistics is unknown. Anderson et al. [12] extended the group sequential tests to the weighted mixed tests for settings where for some (or all) test statistics the correlation is known. Finally, in the present paper, these approaches are extended to adaptive tests, allowing for adaptations such as the selection of hypotheses, or sample size reassessment after an unblinded interim analysis. Table 1 gives an overview of the available fixed sample, group sequential, and adaptive group sequential multiple testing procedures.

**TABLE 1:** Overview of proposed fixed sample and group sequential multiple testing procedures and adaptive multiple testing procedures based on conditional error rates.

Method	Early rejection	Adaptive	Weights	Nonparametric	Parametric	Mixed
[1, 2, 3]			x	x		
[8]			x		x	
[9]			x	x	x	x
[10, 11]	x		x	x		
[12]	x		x	x	x	x
[13]		x			x	
[14]	x	x	x	x	x^a^	
[15, 16, 17]	x	x			x	
[18, 19, 20]	x^a^	x	x	x		
This paper	x	x	x	x	x	x

^a^ Mentioned as extension in the discussion.

We consider two approaches to construct weighted adaptive multiple testing procedures; one based on the combination test principle and the other based on the conditional error rate principle. In the first approach, adaptive p value combination tests [21], in conjunction with closed testing, are used to construct adaptive multiple testing procedures that control the FWER by combining adjusted stage‐wise p values [22, 23]. We consider tests where the adjusted p values are based on weighted Bonferroni tests if the correlation between test statistics is unknown [1], weighted parametric tests if the correlation is known [8], or weighted mixed tests if the correlation between only some of the test statistics is known [9].

The second approach is based on the conditional rejection principle [24, 25, 26]. For settings where the correlation between test statistics is known, this approach has been used to construct multiple testing procedures such as adaptive Dunnett tests [13], adaptive multiarm multistage group sequential designs incorporating early stopping [15, 16], and adaptive multiple testing procedures for clinical trials with subgroup selection [17]. However, these approaches use unweighted tests. For settings where the correlation is unknown, weighted adaptive tests based on partial conditional error rates have been proposed by [18, 19, 20], who generalize the adaptive, fixed sample graph‐based tests to adaptive tests for this scenario. Glimm et al. [14] extended this approach to adaptive tests with early rejection of null hypotheses and discuss an extension to the case where the correlations are known.

In this paper, we extend both the combination approach and the conditional error approach to two‐stage group sequential weighted adaptive tests for scenarios where the correlation may be known between some test statistics and unknown in others. Nonnegative weights can be defined by graphs [1, 2, 3] or, alternatively, arbitrary nonnegative weights summing up to one (or a lower value) can be specified. As the multiple testing procedure is based on the closed testing principle [7], it strongly controls the FWER—not only under the global null hypothesis, where all null hypotheses are true, but also under any partial null, where some null hypotheses may be false.

Furthermore, the considered adaptive approaches do not require prespecification of the adaptation rule to ensure control of the FWER. This contrasts with another class of adaptive designs, in which FWER control is achieved by calculating the type 1 error rate—either analytically or via simulation—under a fixed adaptation rule, and then selecting critical values to match the nominal level [27, 28, 29]. To ensure strong FWER control in such designs, either the worst‐case configuration of true and false null hypotheses (maximizing the type 1 error rate) must be identified or a sufficiently large and representative set of scenarios must be evaluated [19].

Section 2 is a quick review of the graph‐based methodology in [1] and [8], applied to a single stage non adaptive design involving two treatment arms, a common control arm, and two endpoints. Section 3 provides the mathematical notation and technical details for a general two‐ stage graph‐based adaptive design involving k hypotheses. This section is split into two parts: Section 3.1 covers the p value combination method, while Section 3.2 covers the conditional error rate method. Within each of these sections is an illustrative example explaining how to perform the actual computations for the interim and final analyses. Section 4 presents the results of a simulation study that demonstrates FWER control and compares the disjunctive and conjunctive power of the two methods over a range of scenarios and decision rules for selecting hypotheses at the end of stage one. In Section 5, we review our major findings, one of which is that the conditional error approach outperforms the combination approach with respect to both disjunctive and conjunctive power across all scenarios and decision rules considered. We discuss the role that consonance might be playing in this important result. We end with some suggestions for further work.

The flexibility to adaptively alter the future course of a confirmatory clinical trial while preserving its FWER has evolved considerably since the early days of the two arm group sequential design where early stopping for efficacy or futility were the only options. The present work incorporates all the important subsquent advances including sample size re‐estimation, multiple treatment arms, multiple endpoints, treatment or endpoint selection, and graph‐based weighting strategies for hypothesis testing. The incorporation of the graph based methodology into the multiarm group sequential framework requires careful explanation, which has added to the length of the paper and the complexity of the notation.

## 2 Nonadaptive Graph‐Based Closed Testing

Consider the schizophrenia trial introduced in Section 1. Let $\mu_{0,\text{tot}}$ be the mean week‐12 change from baseline in the total PANSS score for the control arm and $\mu_{0,\text{neg}}$ be the mean week‐12 change from baseline in the negative symptoms PANSS score for the control arm. Similarly, let $\mu_{500,\text{tot}}$ be the mean week‐12 change from baseline in the total PANSS score for the 500 mg arm and $\mu_{500,\text{neg}}$ be the mean week‐12 change from baseline in the negative symptoms PANSS score for the 500 mg arm. Finally let $\mu_{350,\text{tot}}$ be the mean week‐12 change from baseline in the total PANSS score for the 350 mg arm and $\mu_{350,\text{neg}}$ be the mean week‐12 change from baseline in the negative symptoms PANSS score for the 350 mg arm. Then $\delta_{1}=\mu_{500,\text{tot}}-\mu_{0,\text{tot}}$ is the treatment effect of the high dose arm for the primary endpoint, $\delta_{2}=\mu_{350,\text{tot}}-\mu_{0,\text{tot}}$ is the treatment effect of the low dose arm for the primary endpoint, $\delta_{3}=\mu_{500,\text{neg}}-\mu_{0,\text{neg}}$ is the treatment effect of the high dose arm for the secondary endpoint and $\delta_{4}=\mu_{350,\text{neg}}-\mu_{0,\text{neg}}$ is the treatment effect of the low dose arm for the secondary endpoint.

**FIGURE 1:**
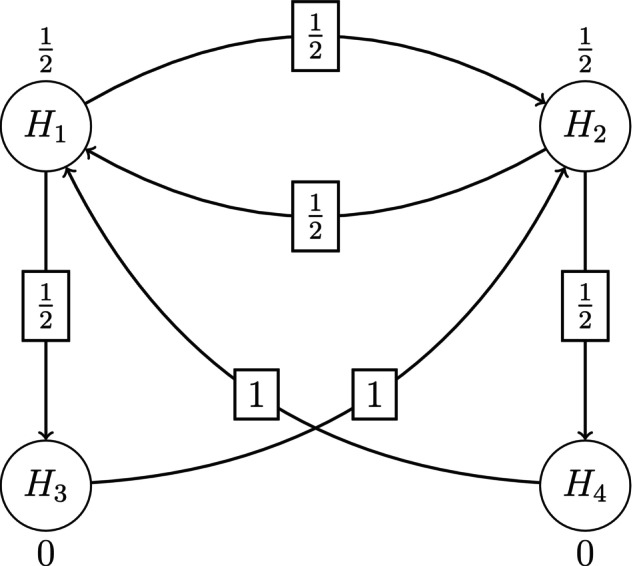
Example of graph‐based weighting strategy proposed by [8].

The investigators are interested in testing each elementary hypothesis $H_{j}\text{:}\;\delta_{j}=0$ against the corresponding one‐sided alternative hypothesis $\delta_{j}>0$, for allj∈I={1,2,3,4} while preserving strong control of the FWER at level α. This can be achieved by carrying out a closed test, which involves testing the intersection hypotheses $H_{J}=\cap_{j\in J}H_{j}$, for all subsets J⊆I, with local level‐α tests. From the perspective of the investigators, however, some hypotheses are more important than others. Therefore it is desirable to conduct weighted hypothesis tests with weights $w_{j,J},j\in J$ assigned to the components of each intersection hypothesis $H_{J}$ for all J⊆I. It is easy to show that there are $|I|\times 2^{\left(|I|-1\right)}=32$ individual weights to be assigned to the full closure tree of the four elementary hypotheses in I. Assigning these weights so that they reflect the study team's relative priorities, and so that these priorities can be easily communicated to trial investigators, poses a challenge. The graph‐based weighting strategy proposed by [8] is an extremely convenient way to achieve both goals. Figure 1 is an example of such a strategy.

It consists of four nodes, one for each elementary hypothesis $H_{j},j\in I$, connected to each other in a specific way by directed edges. A nodal weight $w_{j,I}$ is assigned to each node $H_{j},j\in I$. The edges of the graph are specified through a transition matrix $G=(g_{ij})\leq 1$ connecting the node $H_{i}$ to the node $H_{j}$ with edge weights $0\leq g_{ij},g_{ii}=0$ and $\sum_{j=1}^{\left|I\right|}g_{ij}\leq 1$, for all i,j∈I. In Figure 1 the nodal weights are, $w_{1,I}=w_{2,I}=1/2$ and $w_{3,I}=w_{4,I}=0$. While these are the assigned weights for testing the intersection hypothesis $H_{I}=\cap_{j\in I}H_{j}$, the choice of test may differ, depending on what is known about the distribution of the marginal p values $p_{j},j\in I$. For instance if the correlations among the p values are completely unknown, one might choose the weighted Bonferroni test, which rejects $H_{I}$ if $\cup_{j\in I}[p_{j}\leq w_{j,I}\alpha ]$. On the other hand if the correlations among the p values are known, the level‐α weighted Dunnett test, which rejects $H_{I}$ if $\cup_{j\in I}[p_{j}\leq c_{I}w_{j,I}\alpha ]$, where $c_{I}$ satisfies the relationship $P_{H_{I}}\{\cup_{j\in I}[P_{j}\leq c_{I}w_{j,I}\alpha ]\}=\alpha$, would be more powerful. A testing strategy can also be constructed for the mixed case in which the correlations are known for some $p_{j},j\in I$, but not for others. In effect, we have separated the process of assigning weights to the individual components of the intersection hypothesis $H_{I}$ from the process of determining the appropriate weighted hypothesis test with which to test $H_{I}$.

Returning to Figure 1, the transition matrix is given by

$$G=\left(\begin{array}{cccc} 0 & 1/2 & 1/2 & 0\\ 1/2 & 0 & 0 & 1/2\\ 0 & 1 & 0 & 0\\ 1 & 0 & 0 & 0 \end{array}\right)$$

where, as we shall see shortly, the edge weight $g_{ij}$ represents the fraction of the nodal weight at node $H_{i}$ that would be transferred to node $H_{j}$ if $H_{i}$ were removed from the graph. If $g_{ij}=0$ then the nodes $H_{i}$ and $H_{j}$ are not connected.

Although only the weights for testing the intersection hypothesis $H_{I}$ are displayed explicitly in Figure 1, the weights for all the other intersection hypotheses $H_{J},J\subseteq I$ are actually embedded within it and can be extracted with the help of the transition matrix G. For any fixed J⊆I, this is achieved by selectively removing each node from I∖J, reconnecting the resulting loose edges, and updating the transition matrix, by means of Algorithm 1 of [8]. When all the nodes in I∖J have been thus removed, we are left with a graph whose nodal values $\left\{w_{j,J},j\in J\right\}$ are the desired weights for the intersection hypothesis $H_{J}$. For completeness, we reproduce Algorithm 1 of [8] above.

ALGORITHM 1 **of [8] for extracting the weights $\left\{w_{j,J},j\in J\right\}$ for any intersection hypothesis $H_{J},J\subseteq I$, embedded in a graph specified by initial weights $\left\{w_{j,I},j\in I\right\}$ and transition matrix $G=(g_{ij}),i,j\in I$.**

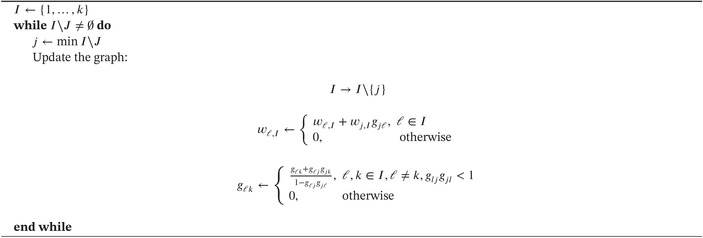

**FIGURE 2:**
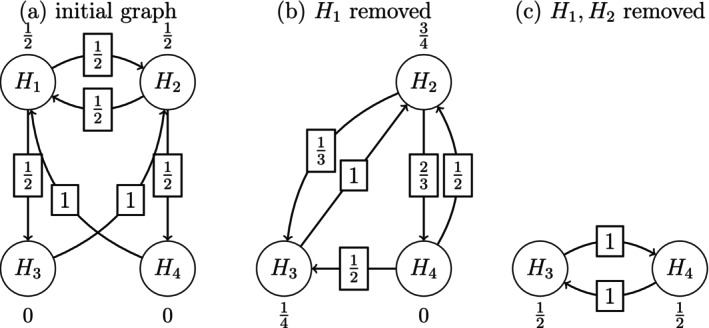
Impact of selective removal of $H_{1}$ and $H_{2}$ from original graph.

This algorithm must be repeated for all subsets J∈I. By way of illustration, Figure 2b displays the resultant graph when the node $H_{1}$ is removed from the initial graph and Figure 2c displays the resultant graph when, in addition, the node $H_{2}$ is removed.

When $H_{1}$ is removed, the resultant graph provides the weights for the intersection hypothesis $H_{J}$, J={2,3,4}, as $w_{2,J}=3/4,w_{3,J}=1/4,w_{4,J}=0$. When, additionally, $H_{2}$ is removed, the resultant graph provides the weights for the intersection hypothesis $H_{J},J=\{3,4\}$, as $w_{3,J}=1/2,w_{4,J}=1/2$. Table 2 displays all 32 weights for the weighting strategy implied by Figure 1.

**TABLE 2:** Weighting Strategy implied by Figure 1.

Index set (J)	Intersection hypothesis ($H_{J}$)	Weights $\left\{w_{j,J},j\in J\right\}$
{1,2,3,4}	$H_{1}\cap H_{2}\cap H_{3}\cap H_{4}$	{0.5, 0.5, 0, 0}
{2,3,4}	$H_{2}\cap H_{3}\cap H_{4}$	{0.75, 0.25, 0}
{1,3,4}	$H_{1}\cap H_{3}\cap H_{4}$	{0.75, 0, 0.25}
{1,2,4}	$H_{1}\cap H_{2}\cap H_{4}$	{0.5, 0.5, 0}
{1,2,3}	$H_{1}\cap H_{2}\cap H_{3}$	{0.5, 0.5, 0}
{3,4}	$H_{3}\cap H_{4}$	{0.5, 0.5}
{2,4}	$H_{2}\cap H_{4}$	{1, 0}
{2,3}	$H_{2}\cap H_{3}$	{0.75, 0.25}
{1,4}	$H_{1}\cap H_{4}$	{0.75, 0.25}
{1,3}	$H_{1}\cap H_{3}$	{1, 0}
{1,2}	$H_{1}\cap H_{2}$	{0.5, 0.5}
{4}	$H_{4}$	{1}
{3}	$H_{3}$	{1}
{2}	$H_{2}$	{1}
{1}	$H_{1}$	{1}

We have shown that the graph $\left\{w_{j,I},j\in I\right\}$, together with the transition matrix $G(I)=\{(g_{ij}),i,j\in I\}$, provides the weights for all the weighted intersection hypotheses constituting the closed test of the elementary hypotheses $H_{j},j\in I$. Since different graphs would give rise to different weighting strategies, it may be asked why the graph displayed in Figure 1, and not any other, should be used for testing the endpoints in the Schizophrenia trial. The reason is that Figure 1 reflects the priorities of the study team. Figures 1 and 2 bear a visual resemblance to the sequentially rejective hierarchical testing scheme of [1]. In that interpretation the total α is split equally between the two doses with a strict hierarchy within dose for testing the secondary endpoint only if the primary endpoint is first rejected. The edge values show that if a primary endpoint is rejected for a given dose, half its share of α is passed down to the corresponding secondary endpoint for that dose, and half to the primary endpoint for the other dose. If both primary and secondary hypotheses are rejected for a given dose, the accumulated α is passed on for testing the primary endpoint of the other dose. When a hypothesis is rejected, the corresponding node is removed from the graph and the graph is updated in accordance with Algorithm 1 of [1] (which is different from Algorithm 1 of [8]). Testing continues until no hypothesis can be rejected. Bretz et al. [1] have shown that the end result of the sequentially rejective testing is the same regardless of the order in which the nodal hypotheses are tested.

This implementation is a completely equivalent short cut for the weighted closed testing procedure described here provided the weighted tests for the intersection hypotheses are consonant. Here, consonance refers to the property that, if the global intersection null hypothesis is rejected, then at least one of the elementary hypotheses is also rejected by the closed testing procedure [30]. If the tests for the intersection hypotheses are not consonant, the weights of the intersection hypotheses elicited by Algorithm 1 of [8] nevertheless result in a valid closed test, but it can no longer be implemented as a sequentially rejective hypothesis testing procedure. One can, however, by analogy with the sequentially rejective testing procedure, interpret the graph as reflecting the priorities of the study team with the nodal weights of the initial graph representing the relative importance of the elementary hypotheses and the edge weights representing the fraction of the nodal weight being transmitted from a source node to a successor node. It is thus both natural and convenient to incorporate and communicate the weighting strategy for a clinical trial with multiple endpoints through a graph. Bretz et al. [1] provide examples of several graphs, each reflecting the different priorities of the study team for the multiple endpoints being tested.

Note that the tests defined by graphs as in [8] can often be made consonant by minor modifications of the graph. Specifically, to ensure consonance, the weights reallocated to any other endpoint upon rejection must not be too small.

So far, we have reviewed the graph‐based multiple testing for a single‐look nonadaptive trial. We will now generalize the testing procedure to two‐stage adaptive designs in which there is α spending for early identification of effective endpoints, endpoint selection, sample size re‐estimation, and alteration of the initial graph based on an unblinded analysis of the stage one data.

## 3 Generalization to Two‐Stage Adaptive Designs

Assume that a set of k>1 one‐sided elementary null hypotheses $H_{i}$, $i\in I_{1}$, $I_{1}=\{1,\ldots ,k\}$ is to be tested with strong control of the FWER at level α over the two stages of an adaptive clinical trial. The hypotheses $H_{i}$ may correspond to different treatments, subgroups, endpoints, or a combination of these. In order to test all these hypotheses with strong FWER control, it is necessary to implement a closed testing procedure. This is achieved by testing all the intersection hypotheses $H_{J},J\subseteq I_{1}$ with local level‐α tests. Moreover, since some hypotheses might be more important than others, weights $\left\{w_{j,J},j\in J\right\}$ will be applied to the individual components of each intersection hypothesis $H_{J}$. A natural and transparent way to specify these weights is through a graph‐based weighting strategy as described in Section 2. Accordingly, we assume that an initial graph defined by weights $\left\{w_{j,I_{1}},j\in I_{1}\right\}$ and transition matrix $\left\{G(I_{1})=(g_{ij}),i,j\in I_{1}\right\}$ has been specified. All $k\times 2^{k-1}$ weights associated with the full closure tree of the k elementary hypotheses indexed by $I_{1}$ can be extracted from the graph with the help of Algorithm 1 of [8].

Having specified the weights for all the intersection hypothesis tests $H_{J},J\subseteq I_{1}$, the next step is to specify the actual hypothesis tests to which these weights will be applied. In an adaptive design, these hypothesis tests are specified in the planning phase, before any actual data from the trial are available. Then, at the end of stage one, on the basis of an unblinded interim analysis, these tests may undergo adaptive modifications. The weighting stategy too may be modified through a revised graph, such that new weights $\left\{{\tilde{w}}_{j,J},j\in J\right\}$ are applied for stage two testing. Additional stage two data are then gathered and a final analysis is performed using all available data over both stages. Even though a closed test will be performed for the final analysis, these adaptive modifications could undermine strong FWER control unless appropriate adjustments are made to the testing procedure. In this paper we will present two adjustment methods, the p value combination method, and the conditional error rate method, and will then compare their operating characteristics. The p value combination method preserves the FWER by combining, for each intersection hypothesis, the p value of the stage one data with the adapted p value of the incremental stage two data using a pre‐specified combination function. The conditional error rate method is based on a principle of [31] which states that if a test is adapted at stage one, its probability of rejection under the null hypothesis, conditional on the observed stage one data, must not exceed the corresponding conditional rejection probability (CER) of the original unadapted test. In settings where the correlation between p values of the individual hypotheses constituting an intersection hypothesis is unknown, the CER cannot be computed. In these cases, we use an extension that controls the sum of partial conditional errors (PCER) [18, 19]. We next discuss these two procedures in detail. Throughout, we will use the notational convention that $p_{J,2}$ is a p value for the test of $H_{J}$ based on the cumulative data from stages one and two, whereas $p_{J,(2)}$ is a p value for the test of $H_{J}$ based only on the incremental data obtained from stage two. Also $P_{J,2}$ and $P_{J,(2)}$ are random variables, whereas $p_{J,2}$ and $p_{J,(2)}$ are specific values attained by them.

### 3.1 The P Value Combination Method

In this method, a combination test is prespecified for each intersection hypothesis $J\subseteq I_{1}$. At the end of stage one, some intersection hypotheses are rejected. The trial proceeds to stage two, possibly after undergoing an adaptive modification, and the remaining intersection hypotheses are tested. The FWER of the individual hypotheses in $I_{1}$ is preserved by a closed test. This is shown below in detail.

#### 3.1.1 Preplanned Tests for the Intersection Hypotheses

In the planning phase of a two‐stage adaptive trial, for each $H_{J},J\subseteq I_{1}$, we specify a level α combination test ${𝒞}_{J}$ with the decision function

(1) $$\varphi_{{𝒞}_{J}}(p_{J,1},p_{J,(2)})=\left\{\begin{array}{ll} 1\; & \text{if}\;p_{J,1}\leq \alpha_{J,1}\;\text{or}\\ \; & \;p_{J,1}>\alpha_{J,1}\;\text{and}\;{𝒞}_{J}(p_{J,1},p_{J,(2)})\leq \alpha_{J,2}\\ 0\; & \text{otherwise} \end{array}\right.$$

where $p_{J,1}$ and $p_{J,(2)}$ are weighted‐adjusted p values for testing $H_{J}$, based on the stage one and incremental stage two data, respectively, $𝒞(p_{J,1},p_{J,(2)})$ is a p value combination function that combines the independent adjusted p values of the two stages into a combined adjusted p value incorporating the data from both stages, and $\left(\alpha_{J,1}\;\text{and}\;\alpha_{J,2}\right)$ are selected so as to satisfy the level α condition

(2) $$P_{H_{J}}(P_{J,1}\leq \alpha_{J,1})+P_{H_{J}}\{P_{J,1}>\alpha_{J,1}\;\text{and}\;{𝒞}_{J}(P_{J,1},P_{J,(2)})\leq \alpha_{J,2}\}=\alpha$$

In this investigation, we will use the inverse normal p value combination function defined by

(3) $${𝒞}_{J}(p_{J,1},p_{J,(2)})=1-\Phi \{\nu_{J,1}\Phi^{-1}(1-p_{J,1})+\nu_{J,2}\Phi^{-1}(1-p_{J,(2)})\}$$

in which $\nu_{J,1}$ and $\nu_{J,2}$ are prespecified constants with $\nu_{J,1}^{2}+\nu_{J,2}^{2}=1$, although other combination functions (for example [21]) could be used as well. While the constants $\nu_{J,1}$ and $\nu_{J,2}$ could depend on J, they are usually selected in proportion to the preplanned sample sizes of the two stages of the trial. They must, however, remain fixed, even if the stage two sample size is adaptively altered.

The formula for computing the weighted‐adjusted p values $p_{J,1}$ and $p_{J,(2)}$ will depend on the available information about the correlations among the p values of the elementary hypotheses constituting $H_{J}$. Consider, for example, the intersection hypotheses displayed in Table 2 for the schizophrenia trial described in Section 2. For some index sets J no information is available. This is the case, for example, for the intersection hypothesis $H_{J}$ when J={1,3} in Table 2, since the correlation between the p values for the primary and secondary endpoints of the schizophrenia trial is unknown. For other index sets the correlations amongst the p values are completely known. This is the case, for example, for J={1,2} in Table 2. Here the correlation between the p values of the two treatment arms of the schizophrenia trial can be deduced from the correlation between their inverse normal transformations, which depends on the allocation ratio of each treatment arm to the placebo arm. Thus, if we set $Z_{1,1}=\Phi^{-1}(1-P_{1,1})$ and $Z_{2,1}=\Phi^{-1}(1-P_{2,1})$ then, for balanced randomization, $\text{corr}(Z_{1,1},Z_{2,1})=0.5$. And by the same reasoning $\text{corr}(Z_{1,(2)},Z_{2,(2)})=0.5$. Finally, there is the mixed case in which the correlations are known for some elements of J and unknown for others. This is the case, for example, for J={1,2,3} in Table 2. As a result, the weighted‐adjusted p values for the intersection hypotheses $H_{J},J\subseteq I_{1}$ are of three types; nonparametric, parametric, and mixed.

 
**Nonparametric** If the correlations among the elementary p values constituting the intersection hypothesis $H_{J}$ are all unknown, compute the weighted Bonferroni‐adjusted p value from the stage one data as
$$p_{J,1}=\min\left(1,\underset{j\in J}{\min}\left\{\frac{p_{j,1}}{w_{j,J}}\right\}\right)$$
  and from the incremental stage two data as
$$p_{J,(2)}=\min\left(1,\underset{j\in J}{\min}\left\{\frac{p_{j,(2)}}{{\tilde{w}}_{j,J}}\right\}\right)$$
 
**Parametric** If the correlations among the p values constituting the intersection hypothesis $H_{J}$ are all known, compute the weighted parametric‐ adjusted p value from the stage one data as
$$p_{J,1}=P_{H_{J}}\left(\underset{j\in J}{\min}\left\{\frac{P_{j,1}}{w_{j,J}}\right\}\leq \underset{j\in J}{\min}\left\{\frac{p_{j,1}}{w_{j,J}}\right\}\right)$$
  and from the incremental stage two data as
(4) $$p_{J,(2)}=P_{H_{J}}\left(\underset{j\in J}{\min}\left\{\frac{P_{j,(2)}}{{\tilde{w}}_{j,J}}\right\}\leq \underset{j\in J}{\min}\left\{\frac{p_{j,(2)}}{{\tilde{w}}_{j,J}}\right\}\right)$$
  In the above formulae if $w_{j,J}$ or ${\tilde{w}}_{j,J}$ is zero for any j∈J, the corresponding weighted p value term is dropped from both sides of the inequality.
 
**Mixed** If the correlations among the elementary p values constituting the intersection hypothesis $H_{J}$ are unknown, for some j∈J and known for others, partition J into l distinct index sets $J=\cup_{h=1}^{l}J_{h}$ such that the correlations among the elementary p values constituting each index set $J_{h},h=1,\ldots l,$ are all known. Then the weighted‐adjusted p value for the mixed case is
$$\begin{array}{l} p_{J,1}=\min\left\{1,\underset{h=1,\ldots ,l}{\min}\left[\frac{P_{H_{J_{h}}}\left(\underset{j\in J_{h}}{\min}\left\{\frac{P_{j,1}}{w_{j,J}}\right\}\leq \underset{j\in J_{h}}{\min}\left\{\frac{p_{j,1}}{w_{j,J}}\right\}\right)}{\sum_{j\in J_{h}}w_{j,J}}\right]\right\} \end{array}$$
  based on the stage one data, and is
(5) $$\begin{array}{ll} p_{J,(2)} & =\min\left\{1,\underset{h=1,\ldots ,l}{\min}\left[\frac{P_{H_{J_{h}}}\left(\underset{j\in J_{h}}{\min}\left\{\frac{P_{j,(2)}}{{\tilde{w}}_{j,J}}\right\}\leq \underset{j\in J_{h}}{\min}\left\{\frac{p_{j,(2)}}{{\tilde{w}}_{j,J}}\right\}\right)}{\sum_{j\in J_{h}}{\tilde{w}}_{j,J}}\right]\right\} \end{array}$$
  based on the incremental stage two data. Note that one or more of the distinct subsets $J_{h}$ partitioning the index set J can be singletons. The above formulae nevertheless remain applicable with the understanding that if the weight $w_{j,J}$ or ${\tilde{w}}_{j,J}$ is zero for any singleton, that term it is dropped from the above probability expression.

#### 3.1.2 Closed Testing at the Interim and Final Analyses

Closed testing requires that every intersection hypothesis $H_{J},J\subseteq I_{1}$ should be tested with a local level‐α test. At the interim, analysis, after the stage one data have been observed, all the intersection hypotheses $H_{J},J\subseteq I_{1}$, that satisfy $p_{J,1}\leq \alpha_{J,1}$ are rejected. For all $J\subseteq I_{1}$, let 𝒫(J) denote the set of all non‐empty subsets of J. That is, 𝒫(J) is the power set of J. Then ${𝒥}^{r}=\{J\in 𝒫(I_{1})|p_{J,1}\leq \alpha_{J,1}\}$ denotes the index set of intersection hypotheses rejected at the interim analysis. Since the status of these intersection hypotheses has been determined at stage one, they need not be considered for further testing at stage two. It remains to determine, at stage two, the status of the remaining intersection hypotheses, indexed by ${𝒥}^{+}=𝒫(I_{1})\setminus \{{𝒥}^{r}\}$, for which no test decision was made at the end of stage one. However, stage two data might not be available for computing adjusted stage two p values $p_{J,(2)}$ for all the $H_{J}$ with $J\in {𝒥}^{+}$. To see this, we first observe that all individual hypotheses $H_{i},i\in I_{1}$, for which all intersection hypotheses $H_{J},J\subseteq I_{1}$ with i∈J, are in ${𝒥}^{r}$, are rejected by the closed test. Denote the index set containing these individual hypotheses by $I_{1r}\subseteq I_{1}$. These hypotheses, having demonstrated efficacy at stage one itself, need not be tested again at stage two. Therefore, stage two data would be available, potentially, only for testing the remaining individual hypotheses $I_{1}^{*}=I_{1}\setminus \{I_{1r}\}$. In an adaptive trial, however, there is also the option to drop additional elementary hypotheses from $I_{1}^{*}$. Suppose then that, after an unblinded examination of the stage one data, it is decided to select only a subset $I_{2}\subseteq I_{1}^{*}$ for stage two testing and to drop the remaining $I_{1}^{*}\setminus I_{2}$ hypotheses from further consideration without gathering their stage two data. The rationale for dropping some hypotheses from $I_{1}^{*}$ without stage two testing might be concerns about safety, efficacy, cost, enrollment, or other issues unrelated to formal statistical testing. The adaptive procedure allows full flexibility to decide which hypotheses in $I_{1}^{*}$ to drop and which ones to select for stage two testing. There is further flexibility to reassess and alter, if necessary, the stage two sample size and the graph‐based representation of the priorities for testing the hypotheses indexed by $I_{2}$. After making all these adaptive changes, the trial proceeds to stage two for computing the adjusted p values $p_{J,(2)},J\in {𝒥}^{+}$, with incomplete stage two data for some $H_{J}$. Notwithstanding the missing data, these adjusted p values must be specified since closed testing requires that $H_{J},J\in {𝒥}^{+}$ be tested with local level‐α tests for all $H_{J},J\in {𝒥}^{+}$. To that end we partition ${𝒥}^{+}$ into three nonoverlapping subsets ${𝒥}_{A},{𝒥}_{B}$ and ${𝒥}_{C}$ depending on the manner in which the adjusted p values $p_{J,(2)}$ are specified in each subset.

 
${𝒥}_{A}=𝒫(I_{2})\cap {𝒥}^{+}:$ Since the elementary hypotheses $H_{i}$, $i\in I_{2}$ will be undergoing stage two testing, p values $p_{i,(2)},i\in I_{2}$ will be available for each of them, and hence adjusted p values for all intersection hypotheses $H_{J},J\in 𝒫(I_{2})$ can be derived.
 
${𝒥}_{B}=𝒫(I_{1}^{*}\setminus I_{2})\cap {𝒥}^{+}:$ Since the elementary hypotheses $H_{i}$, $i\in I_{1}^{*}\setminus I_{2}$ will not have any stage two data, neither will the intersection hypotheses $H_{J},J\in {𝒥}_{B}$. Therefore we set $p_{J,(2)}=1$ for all $J\in {𝒥}_{B}$.
 
${𝒥}_{C}={𝒥}^{+}\setminus \{{𝒥}_{A}\cup {𝒥}_{B}\}:$ Since the intersection hypotheses $H_{J},J\in {𝒥}_{C}$ belong neither to ${𝒥}_{A}$ nor to ${𝒥}_{B}$, it must be the case that for all $J\in {𝒥}_{C}$, $J\cap I_{2}\neq J$ and $J\cap I_{2}\neq \emptyset$. Therefore we will set the stage two p value as
(6) $$p_{J,(2)}=p_{J\cap I_{2},(2)}$$
  for all $J\in {𝒥}_{C}$. Since $J\cap I_{2}\in {𝒥}_{A}$, these stage two p values can be obtained from ${𝒥}_{A}$.

Adjusted p values $p_{J,1}$ and $p_{J,(2)}$ are thus available for all $J\in {𝒥}^{+}$ at the end of stage 2, so that the combination test (1) can be evaluated for all $J\in \{{𝒥}^{+}\cup {𝒥}^{r}\}=𝒫(I_{1})$. Finally, applying the closed testing principle, a hypothesis $H_{i},i\in I_{2}$, can be rejected at familywise error rate α if all intersection hypotheses $H_{J},J\subseteq I_{1}$ with i∈J, are rejected with their respective level α combination tests. Thus for all $i\in I_{2}$ the decision function of the closed test of $H_{i}$ is given by

(7) $$\varphi_{i}=\underset{\left\{J\in 𝒫(I_{1}),\;i\in J\right\}}{\min}\varphi_{C_{J}}(p_{J,1},p_{J,(2)})$$

#### 3.1.3 Illustrative Example of the P Value Combination Method

Returning to the schizophrenia trial specified at the beginning of Section 2, suppose that the total sample size is 210 subjects with a balanced randomization of 70 subjects per arm. It is easy to show that with this sample size a single stage one sided level‐0.025 Dunnett test has 89% disjunctive power to detect a mean improvement of 10 units in the total PANSS score (given σ=20). Now let $I_{1}=\{1,2,3,4\}$ denote the index set of the four elementary hypotheses, and let the weighting strategy for testing all the intersection hypotheses $H_{J},J\subseteq I_{1}$ be represented graphically by Figure 1, and in tabular form by Table 2. We will assume that there is an interim look after 50% of the subjects have enrolled, and the trial spends the available one sided α=0.025 over the two stages of the trial in accordance with the [32] O'Brien‐Fleming (LDOF) type error spending function

(8) $$g(t,\alpha )=2-2\Phi \left(\frac{z_{\alpha /2}}{\sqrt{t}}\right)$$

where t is the information fraction at the end of stage one. Since t=0.5, by (8) the amount of α available for testing each $H_{J}$ at the end of stage one is $\alpha_{J,1}=0.00153$. Suppose that at the end of stage one the marginal p values for the elementary hypotheses are $p_{1,1}=0.00045,p_{2,1}=0.0952,p_{3,1}=0.0225$ and $p_{4,1}=0.1104$. These four marginal p values are the building blocks for generating the weighted‐adjusted p value $p_{J,1}$, with the weights $w_{J}=\{w_{j,J},j\in J\}$ displayed in Table 2, for testing the intersection hypothesis $H_{J}$ for all $J\subseteq I_{1}$. Since $\alpha_{J,1}=0.00153$, any $H_{J}$ for which $p_{J,1}\leq 0.00153$ will be rejected at stage one.

Table 3 extends Table 2 by including two new columns containing, respectively, the weighted‐adjusted p value and the corresponding adjustment method for all the intersection hypotheses $H_{J},J\subseteq I_{1}$ derived from $p_{1,1}=0.00045,p_{2,1}=0.0952,p_{3,1}=0.0225,p_{4,1}=0.1104$.

**TABLE 3:** Weighted‐adjusted p values based on stage one marginal p values. Note that for the computation of the adjusted p values only the correlation between test statistics of hypotheses $H_{j}$ with $w_{j,J}\neq 0$ needs to be known.

Index set	Intersection hypothesis	Weights	Adjusted p value	Adjustment
(J)	($H_{J}$)	$\left\{w_{j,J},j\in J\right\}$	$p_{J,1}$	method
{1,2,3,4}	$H_{1}\cap H_{2}\cap H_{3}\cap H_{4}$	{0.5, 0.5, 0, 0}	0.00088	Parametric
{2,3,4}	$H_{2}\cap H_{3}\cap H_{4}$	{0.75, 0.25, 0}	0.0900	Nonparametric
{1,3,4}	$H_{1}\cap H_{3}\cap H_{4}$	{0.75, 0, 0.25}	0.0006	Nonparametric
{1,2,4}	$H_{1}\cap H_{2}\cap H_{4}$	{0.5, 0.5, 0}	0.00088	Parametric
{1,2,3}	$H_{1}\cap H_{2}\cap H_{3}$	{0.5, 0.5, 0}	0.00088	Parametric
{3,4}	$H_{3}\cap H_{4}$	{0.5, 0.5}	0.0410	Parametric
{2,4}	$H_{2}\cap H_{4}$	{1, 0}	0.0952	NA
{2,3}	$H_{2}\cap H_{3}$	{0.75, 0.25}	0.0900	Nonparametric
{1,4}	$H_{1}\cap H_{4}$	{0.75, 0.25}	0.0006	Nonparametric
{1,3}	$H_{1}\cap H_{3}$	{1, 0}	0.00045	NA
{1,2}	$H_{1}\cap H_{2}$	{0.5, 0.5}	0.00088	Parametric
{4}	$H_{4}$	{1}	0.1104	NA
{3}	$H_{3}$	{1}	0.0225	NA
{2}	$H_{2}$	{1}	0.0952	NA
{1}	$H_{1}$	{1}	0.00045	NA

An examination of Table 3 reveals that the weighted‐adjusted p values of all the intersection hypotheses $H_{J}$ containing $H_{1}$ satisfy $p_{J,1}\leq \alpha_{J,1}=0.00153$. Therefore, the elementary hypothesis $H_{1}$ is rejected by the closed test at stage one. We may thus eliminate the node $H_{1}$ from the initial graph and construct a new graph with updated weights and edges in accordance with Algorithm 1 of [1] as shown in Figure 2b.

Before proceeding to stage two, the trial sponsors re‐consider their priorities in light of $H_{1}$ having been rejected at stage one itself. One option would be to discontinue any further enrollment to the high dose arm and re‐assign the 35 subjects who would have been randomized to that arm during stage two to the remaining two arms. This would increase the power for detecting efficacy in the low‐dose arm for both primary and secondary endpoints. The sponsors conclude, however, that from both a business and patient benefit perspective it is more desirable to continue enrolling patients to all three arms in stage two as originally planned, because of the possibility of claiming efficacy for the secondary endpoint, PANSS for negative symptoms, in the high dose arm.

We note that such a strategy carries the risk that, if $H_{1}$ is tested again at the final analysis, it may no longer be rejected within the closed testing framework. From a statistical perspective, such an additional test is only exploratory in nature and cannot overturn a previous rejection. Nonetheless, a major inconsistency may weaken the credibility of an earlier positive finding and influence regulatory decision‐making (see, e.g., the discussion in section 3.1.2 of [33]).

It is thus decided to select all three hypotheses $H_{2},H_{3}$, and $H_{4}$ for stage two testing so that $I_{2}=\{2,3,4\}$. While the adaptive methodology of closed combination testing would permit an increase in the sample sizes of the three treatment arms and would also permit changes in the hierarchical testing strategy, currently reflected in the Figure 2b, the sponsors have decided to proceed to stage two without making any adaptive changes to the initial design. Accordingly, the study proceeds to stage two with the graphical testing strategy depicted in Figure 2b.

Suppose the unadjusted p values, based on the incremental stage two data, are $\left\{p_{2,(2)}=0.1121,p_{3,(2)}=0.0112,p_{4,(2)}=0.1153\right\}$ respectively for $\left\{H_{2},H_{3},H_{4}\right\}$. We are thus required to test the intersection hypotheses $H_{J}$ for all $J\in {𝒥}^{+}=\{\{2\},\{3\},\{4\},\{2,3\},\{2,4\},\{3,4\},\{2,3,4\}\}$. Multiplicity adjusted weighted p values induced by the above three unadjusted p values are computed in exactly the same way as was done for the stage one p values. Table 4 displays the weighted‐adjusted stage two p values and indicates whether they belong to the parametric, nonparametric, or mixed cases. In this table, however, none of the adjusted p values belongs to the mixed category.

**TABLE 4:** Adjusted p values from incremental stage two data for all intersection hypotheses $H_{J},J\in {𝒥}^{+}$.

Index set	Intersection hypothesis	Weights	Adjusted p value	Adjustment
$J\in {𝒥}^{+}$	$H_{J}$	$\left\{w_{j,J},j\in J\right\}$	$p_{J,(2)}$	method
{2, 3, 4}	$H_{2}\cap H_{3}\cap H_{4}$	0.75, 0.25, 0	0.0448	Nonparametric
{3, 4}	$H_{3}\cap H_{4}$	0.5, 0.5	0.0209	Parametric
{2, 4}	$H_{2}\cap H_{4}$	1.0, 0	0.1121	NA
{2, 3}	$H_{2}\cap H_{3}$	0.75, 0.25	0.0448	Nonparametric
{4}	$H_{4}$	NA	0.1153	NA
{3}	$H_{3}$	NA	0.0112	NA
{2}	$H_{2}$	NA	0.1121	NA

To complete the final analysis, the adjusted p values of the two stages are combined with the prespecified inverse normal combination function

$$\begin{array}{ll} & {𝒞}_{J}(p_{J,1},p_{J,(2)})=1-\Phi \left[\sqrt{\frac{1}{2}}\Phi^{-1}(1-p_{J,1})+\sqrt{\frac{1}{2}}\Phi^{-1}(1-p_{J,(2)})\right] \end{array}$$

for all $J\in {𝒥}^{+}$. The combined p values are displayed in Table 5.

**TABLE 5:** Final p values, based on combining the adjusted p values from the two stages, for all intersection hypotheses $H_{J},J\in {𝒥}^{+}$.

Index set	Intersection hypothesis	Stage‐wise adjusted p values		Combined p value
$J\in {𝒥}^{+}$	$H_{J}$	$p_{J,1}$	$p_{J,(2)}$	${𝒞}_{J}(p_{J,1},p_{J.(2)})$
{2, 3, 4}	$H_{2}\cap H_{3}\cap H_{4}$	0.0900	0.0448	0.0158
{3, 4}	$H_{3}\cap H_{4}$	0.0410	0.0209	0.0038
{2, 4}	$H_{2}\cap H_{4}$	0.0952	0.1121	0.0371
{2, 3}	$H_{2}\cap H_{3}$	0.0900	0.0448	0.0158
{4}	$H_{4}$	0.1104	0.1153	0.0433
{3}	$H_{3}$	0.0225	0.0112	0.0012
{2}	$H_{2}$	0.0952	0.1121	0.0371

The hypothesis $H_{J}$ is rejected at stage two if ${𝒞}_{J}(p_{J,1},p_{J,2})\leq \alpha_{J,2}$ where $\alpha_{J,2}$ satisfies (2). It is easy to show by bivariate normal integration that $\alpha_{J,2}=0.0245$. Then Table 5 shows that $H_{3},H_{2}\cap H_{3},H_{3}\cap H_{4}$ and $H_{2}\cap H_{3}\cap H_{4}$ are all rejected by their local level‐α tests. Thus, $H_{3}$ is rejected by a closed test at stage two.

Suppose that at the end of stage one we decide to adapt the weights associated with the hypotheses in $I_{2}$ from $w_{2,I_{2}}=0.75$, $w_{3,I_{2}}=0.25$, $w_{4,I_{2}}=0$, to ${\tilde{w}}_{2,I_{2}}=0.5$, ${\tilde{w}}_{3,I_{2}}=0.25$, ${\tilde{w}}_{4,I_{2}}=0.25$. By thus assigning a nonzero weight to hypothesis $H_{4}$, we have converted the stage two test of the intersection hypothesis $H_{2}\cap H_{3}\cap H_{4}$ from a nonparametric test to a mixed test in which the index set $I_{2}=\{2,3,4\}$ is partitioned into the union of $J_{1}=\{2\}$ and $J_{2}=\{3,4\}$. For illustrative purposes, we now show how this mixed case intersection hypothesis may be tested. The adjusted p value $p_{I_{2},(2)}$ is obtained from equation (5) rather than from equation (4). Thus

$$\begin{array}{ll} p_{I_{2},(2)} & =\min\left\{1,\frac{1}{0.5}\min\left[P_{H_{2}}\left(\frac{P_{2,(2)}}{0.5}\leq \frac{0.1121}{0.5}\right),\right.\right.\\ & \;\left.\left.P_{H_{3}\cap H_{4}}\left(\min\left(\frac{P_{3,(2)}}{0.25},\frac{P_{4,(2)}}{0.25}\right)\leq \frac{0.0112}{0.25}\right)\right]\right\}\\ & =\min(0.2242,0.0418) \end{array}$$

where the second term is evaluated by converting the probabilities into Z‐statistics by inverting the standard normal CDF and using the pmvnorm function in mvtnorm library to perform the required multivariate normal integration. The changed weights for the hypotheses in $I_{2}$ induce corresponding changes in the weights and adjusted p values for other intersection hypotheses such that Table 4 is replaced by Table 6, and Table 5 is replaced by Table 7.

**TABLE 6:** Impact on Table 4 if weights $\left\{w_{j,I_{2}},j\in I_{2}\right\}$ are adaptively altered from {0.75,0.25,0} to {0.5,0.25,0.25}.

Index set	Intersection hypothesis	Weights	Adjusted p value	Adjustment
$J\in {𝒥}^{+}$	$H_{J}$	$\left\{w_{j,J},j\in J\right\}$	$p_{J,(2)}$	method
{2, 3, 4}	$H_{2}\cap H_{3}\cap H_{4}$	0.5, 0.25, 0.25	0.0418	Mixed
{3, 4}	$H_{3}\cap H_{4}$	0.4165, 0.5835	0.0250	Parametric
{2, 4}	$H_{2}\cap H_{4}$	0.75, 0.25	0.1495	Nonparametric
{2, 3}	$H_{2}\cap H_{3}$	0.625, 0.375	0.0300	Nonparametric
{4}	$H_{4}$	NA	0.1153	NA
{3}	$H_{3}$	NA	0.0112	NA
{2}	$H_{2}$	NA	0.1121	NA

**TABLE 7:** Impact on Table 5 if weights $\left\{w_{{j,I}_{2}},j\in I_{2}\right\}$ are adaptively altered from {0.75,0.25,0} to {0.5,0.25,0.25}.

Index set	Intersection hypothesis	Stage‐wise adjusted p values		Combined p value
$J\in {𝒥}^{+}$	$H_{J}$	$p_{J,1}$	$p_{J,(2)}$	${𝒞}_{J}(p_{J,1},p_{J,(2)})$
{2, 3, 4}	$H_{2}\cap H_{3}\cap H_{4}$	0.0900	0.0418	0.0149
{3, 4}	$H_{3}\cap H_{4}$	0.0410	0.0250	0.0045
{2, 4}	$H_{2}\cap H_{4}$	0.0952	0.1495	0.0484
{2, 3}	$H_{2}\cap H_{3}$	0.0900	0.0300	0.0113
{4}	$H_{4}$	0.1104	0.1153	0.0433
{3}	$H_{3}$	0.0225	0.0112	0.0012
{2}	$H_{2}$	0.0952	0.1121	0.0371

While the table entries differ between corresponding tables, the final outcome for the clinical trial is unchanged. It is still the case that $H_{3}$ is rejected by a closed test at stage two.

### 3.2 The Conditional Error Rate Method

In this method, a two‐stage group sequential level‐α test is prespecified for each intersection hypothesis $H_{J},J\subseteq I_{1}$. At the end of stage one, a conditional error rate (CER), or a sum of partial conditional error rates (PCER), is computed for each intersection hypotheses not rejected early. The trial proceeds to stage two, possibly after undergoing an adaptive modification. The unrejected intersection hypotheses are tested again, this time with modified critical cutoffs that preserve the CER or sum of PCERs, respectively. The FWER of the individual hypotheses in $I_{1}$ is preserved by a closed test. This is shown below in detail.

#### 3.2.1 Preplanned Tests Prior to Stage One

Tests $\varphi_{J}$ are prespecified for all intersection hypotheses $H_{J},J\subseteq I_{1}$. These tests differ depending on whether correlations among the individual p values of the elementary hypotheses constituting $H_{J}$ are all unknown (nonparametric case), all known (parametric case), or partially known (mixed case). Let $p_{j,1}$ be the p value for the elementary hypothesis $H_{j}$ based on stage one data and let

(9) $$p_{j,2}=1-\Phi \left(\sqrt{t}\;\Phi^{-1}(1-p_{j,1})+\sqrt{1-t}\;\Phi^{-1}(1-p_{j,(2)})\right)$$

denote the p value for the elementary hypothesis $H_{j}$ based on the data from both stages, for all $j\in I_{1}$ in the pre‐specified trial. Here, t denotes the preplanned information fraction of the first stage and $p_{j,(2)}$ the p value of the hypothesis test computed based on the second stage data only. Let $\left\{w_{j,J},j\in J\right\}$ be the graph‐based weights assigned to the components of the intersection hypothesis $H_{J}$ for each $J\subseteq I_{1}$.

 
**Nonparametric** Suppose the joint distribution of the $P_{j,i}$ and $P_{j^{\prime },i}$ is unknown for all $j,j^{\prime }\in J,j\neq j^{\prime },i=1,2$, and that $\alpha_{J,1}$ has been preassigned as the amount of type‐1 error to be spent for testing $H_{J}$ at the end of stage one, possibly through pre‐specification of a standard group sequential error spending function. Then the first and second stage critical constants $c_{J,1}$ and $c_{J,2}$ are obtained as solutions to the group sequential equations
(10) $$\underset{j\in J}{\sum }P_{H_{j}}\left\{P_{j,1}\leq w_{j,J}c_{J,1}\right\}=\alpha_{J,1}$$
(11) $$\underset{j\in J}{\sum }P_{H_{j}}\left\{\left[P_{j,1}\leq w_{j,J}c_{J,1}]\;\text{or}\;[P_{j,2}\leq w_{j,J}c_{J,2}\right]\right\}=\alpha$$
  Since the individual p values are assumed to be uniformly distributed and the weights $w_{j,J}$ sum up to one, $c_{J,1}=\alpha_{J,1}$. Now we define the test statistic
(12) $$\phi_{J}=\underset{j\in J}{\sum }I\left\{\left[p_{j,1}\leq w_{j,J}c_{J,1}]\;\text{or}\;[p_{j,2}\leq w_{j,J}c_{J,2}\right]\right\}$$
  where I{·} denotes the indicator function. Note that $\phi_{J}$ corresponds to the number of rejections in the intersection hypothesis test of $H_{J}$. Finally, the decision function $\varphi_{J}=\min(1,\phi_{J})$ defines a conservative two‐stage level α test of $H_{J}$, since
$$\begin{array}{ll} & E_{H_{J}}(\varphi_{J})\leq E(\phi_{J})\\ & \;=\underset{j\in J}{\sum }E_{H_{j}}I\left\{\left[P_{j,1}\leq w_{j,J}c_{J,1}]\;\text{or}\;[P_{j,2}\leq w_{j,J}c_{J,2}\right]\right\}=\alpha \end{array}$$
 
**Parametric** Suppose the joint distribution of $P_{j,i}$ and $P_{j^{\prime },i}$ is known for all $j,j^{\prime }\in J,j\neq j^{\prime },i=1,2$. As in the nonparametric case, let $\alpha_{J,1}$ denote the amount of type‐1 error to be spent for testing $H_{J}$ at the end of stage one. The critical constants $c_{J,1}$ and $c_{J,2}$ are evaluated as solutions to the multiarm group sequential equations
(13) $$P_{H_{J}}\{\cup_{j\in J}[P_{j,1}\leq w_{j,J}c_{J,1}]\}=\alpha_{J,1}$$
(14) $$P_{H_{J}}\{\cup_{j\in J}[P_{j,1}\leq w_{j,J}c_{J,1}]\;\text{or}\;\cup_{j\in J}[P_{j,2}\leq w_{j,J}c_{J,2}]\}=\alpha$$
  These computations could be performed with the help of the R function pmvnorm belonging to the library mvtnorm. The decision function
(15) $$\varphi_{J}=I\left\{\cup_{j\in J}[p_{j,1}\leq w_{j,J}c_{J,1}]\;\text{or}\cup_{j\in J}[p_{j,2}\leq w_{j,J}c_{J,2}]\right\}$$
  defines an exact two‐stage level‐α test of $H_{J}$. That is, $E_{H_{J}}(\varphi_{J})=\alpha$. Note that in the above evaluation of the critical constants $c_{J,1}$ and $c_{J,2}$ all probability expressions in which $w_{j,J}=0$ will be dropped.
 
**Mixed** Suppose that for i=1,2, the joint distribution of $P_{j,i}$ and $P_{j^{\prime },i}$ is known for some $j,j^{\prime }\in J,j\neq j$, and unknown for others. Let J be partitioned into l distinct index sets
(16) $$J=\cup_{h=1}^{l}J_{h}$$
  such that the correlations among the elementary p values constituting each subset $J_{h}$ are known. Note some of these distinct subsets partitioning J can be singletons, implying that the correlations between their p value and the p values belonging to any other subset are unknown. Furthermore, note that the $J_{h}$ depend in addition to h also on the set J under consideration. For notational convenience, however, the dependence of these sets on J has been suppressed. Let $\alpha_{J,1}$ denote the amount of type‐1 error allowable at stage one. Then the critical constants $c_{J,1},c_{J,2}$ are solutions to the group sequential equations
$$\overset{l}{\underset{h=1}{\sum }}P_{H_{J_{h}}}\{\cup_{j\in J_{h}}[P_{j,1}\leq w_{j,J}c_{J,1}]\}=\alpha_{J,1}$$
$$\overset{l}{\underset{h=1}{\sum }}P_{H_{J_{h}}}\{\cup_{j\in J_{h}}[P_{j,1}\leq w_{j,J}c_{J,1}]\;\text{or}\;\cup_{j\in J_{h}}[P_{j,2}\leq w_{j,J}c_{J,2}]\}=\alpha$$
  To construct the test for $H_{J}$, we compute the test statistic
(17) $$\phi_{J}=\overset{l}{\underset{h=1}{\sum }}I\{\cup_{j\in J_{h}}[p_{j,1}\leq w_{j,J}c_{J,1}]\;\text{or}\cup_{j\in J_{h}}[p_{j,2}\leq w_{j,J}c_{J,2}]\}$$
  for the test of $H_{J}$. Finally, $\varphi_{J}=\min(1,\phi_{J})$ defines a conservative two‐stage level α test of $H_{J}$, because $E_{H_{J}}(\varphi_{J})\leq E_{H_{J}}(\phi_{J})=\alpha$. It is easy to show that the mixed case specializes to the nonparametric case if $J_{h},h=1,\ldots ,l$ are all singletons, and specializes to the parametric case if l=1.

#### 3.2.2 Adjustments to Preplanned Tests Due to Stage Two Design Adaptations

At the end of stage one, we identify the sets ${𝒥}^{r},I_{1r},I_{1}^{*}$ and ${𝒥}^{+}$, exactly as defined in Section 3.1.2. Additionally the stage two portion of the trial may be adapted by selecting a subset $I_{2}\subseteq I_{1}^{*}$ of the elementary hypotheses that remain to be tested, altering the sample sizes or allocation ratios of the treatment arms, and changing the weighting strategy for stage two through a revised graph. If the study design undergoes any of these modifications the modified test ${\tilde{\varphi }}_{J}$ may no longer satisfy the level‐α condition

(18) $$E_{H_{J}}({\tilde{\varphi }}_{J})\leq \alpha$$

It is possible, however, to satisfy (18) by imposing on each ${\tilde{\varphi }}_{J},J\in {𝒥}^{+}$, a constraint derived from a generalization of the conditional error rate principle due to [24, 31]. The constraint depends on the stage one data, represented by $\chi_{1}$, and differs depending on whether the test for $\tilde{\varphi }$ is nonparametric, parametric or mixed.

 
**Nonparametric** Define
(19) $$B_{J}(\chi_{1})=\underset{j\in J}{\sum }P_{H_{j}}\{P_{j,2}\leq w_{j,J}c_{J,2}|p_{j,1}\}$$
  as the sum of conditional probabilities for rejecting each individual hypothesis $H_{j}$ as part of the intersection hypothesis $H_{J}$, given the stage one data. We refer to each term in this sum as a partial conditional error rate (PCER). Then (18) is satisfied if the adapted test ${\tilde{\varphi }}_{J}$ is constrained by the condition
(20) $$E_{H_{J}}[{\tilde{\varphi }}_{J}|\chi_{1}]\leq B_{J}(\chi_{1})$$
  uniformly over all possible stage one outcomes $\chi_{1}$. To see this note that the expected value of the statistic (12) can be written as
$$E_{H_{J}}(\phi_{J})=\underset{j\in J}{\sum }E_{H_{j}}\{I[P_{j,1}\leq w_{j,J}c_{J,1}\;\text{or}\;P_{j,2}\leq w_{j,J}c_{J,2}]\}$$
  so that its conditional expectation is
$$E_{H_{J}}(\phi_{J}|\chi_{1})=\underset{j\in J}{\sum }E_{H_{j}}I\{[P_{j,2}\leq w_{j,J}c_{J,2}]|p_{j,1}\}=B_{J}(\chi_{1})$$
  It follows that
$$\begin{array}{ll} E_{H_{J}}({\tilde{\varphi }}_{J}) & =E_{\chi_{1}}E_{H_{J}}[{\tilde{\varphi }}_{J}|\chi_{1}]\leq E_{\chi_{1}}B_{J}(\chi_{1})=E_{\chi_{1}}E_{H_{J}}[\phi_{J}|\chi_{1}]\\ & =E_{H_{J}}(\phi_{J})=\alpha \end{array}$$
  We refer to (20) as the PCER condition. Note that, since $\varphi_{J}\leq \phi_{J}$, the preplanned test satisfies the PCER condition. Thus if, after examining the stage one data, no adaptations are performed, the preplanned testing procedure can be used without any error inflation.
  Operationally, the PCER condition implies that the stage two critical value ${\tilde{c}}_{J,2}$ for the adapted nonparametric test of $H_{J}$ should satisfy the constraint
(21) $$\underset{j\in J}{\sum }P_{H_{j}}\{({\tilde{P}}_{j,2}\leq {\tilde{w}}_{j,J}{\tilde{c}}_{J,2})|(p_{j,1},j\in J)\}\leq B_{J}(\chi_{1})$$
  where (throughout) the ‘tilde’ above a symbol implies that the corresponding term may have been adaptively altered.
 
**Parametric** Define
(22) $$B_{J}(\chi_{1})=P_{H_{J}}\{\cup_{j\in J}[P_{j,2}\leq w_{j,J}c_{J,2}]|(p_{j,1},j\in J)\}$$
  as the conditional error rate (CER), or conditional probability of rejecting $H_{J}$ given the stage one data. Then (18) is satisfied if the adapted test ${\tilde{\varphi }}_{J}$ is constrained by the condition
(23) $$E_{H_{J}}[{\tilde{\varphi }}_{J}|\chi_{1}]\leq B_{J}(\chi_{1})$$
  uniformly over all possible stage one outcomes $\chi_{1}$. To see this note that because
$$E_{H_{J}}(\varphi_{J}|\chi_{1})=E_{H_{J}}\{I[\cup_{j\in J}P_{J,2}\leq w_{j,J}c_{J,2}]|(p_{j,1},j\in J)\}=B_{J}(\chi_{1})$$
  it follows that
$$\begin{array}{ll} E_{H_{J}}({\tilde{\varphi }}_{J}) & =E_{\chi_{1}}E_{H_{J}}[{\tilde{\varphi }}_{J}|\chi_{1}]\leq E_{\chi_{1}}B_{J}(\chi_{1})=E_{\chi_{1}}E_{H_{J}}[\varphi_{J}|\chi_{1}]\\ & =E_{H_{J}}(\varphi_{J})=\alpha \end{array}$$
  We refer to (23) as the CER condition. It was proposed originally by [24, 31] for settings in which $E_{H_{J}}(\varphi_{J}|\chi_{1})$ could be evaluated. Notice that the CER condition is satisfied trivially by the pre‐planned test since $E_{H_{J}}(\varphi_{J}|\chi_{1})=B_{J}(\chi_{1})$. Thus, as with the nonparametric case, one is free to examine the stage one data and revert to the preplanned test at stage two if there are no adaptations.
  Operationally, the CER condition implies that the stage two critical value ${\tilde{c}}_{J,2}$ for the adapted parametric test of $H_{J}$ should satisfy the constraint
(24) $$P_{H_{J}}\{\cup_{j\in J}[{\tilde{P}}_{j,2}\leq {\tilde{w}}_{j,J}{\tilde{c}}_{J,2}]|(p_{j,1},j\in J)\}\leq B_{J}(\chi_{1})$$
 
**Mixed** Partition of the index set J as shown in (16) and compute, for each h=1,2,…l,
$$P_{H_{J_{h}}}\{[\cup_{j\in J_{h}}(P_{j,2}\leq w_{j,J}c_{J,2})]|(p_{j,1},j\in J_{h})\}$$
  the conditional probability to reject any hypothesis $H_{j},j\in J_{h}$ in the test of $H_{J}$. Define
(25) $$B_{J}(\chi_{1})=\overset{l}{\underset{h=1}{\sum }}P_{H_{J_{h}}}\{[\cup_{j\in J_{h}}(P_{j,2}\leq w_{j,J}c_{J,2})]|(p_{j,1},j\in J_{h})\}$$
  as the sum of the above conditional rejection probabilities. We refer to each term in this sum as a partial conditional error rate (PCER). It is in fact the conditional error rate for the parametric test of $H_{J_{h}}$, where $H_{J_{h}}$ is a component of the mixed case intersection hypothesis $H_{J}$. Then (18) is satisfied if the adapted test ${\tilde{\varphi }}_{J}$ is constrained by the condition
(26) $$E_{H_{J}}[{\tilde{\varphi }}_{J}|\chi_{1}]\leq B_{J}(\chi_{1})$$
  uniformly over all possible stage one outcomes $\chi_{1}$. To see this note that the expected value of the statistic (17) can be written as
$$E_{H_{J}}(\phi_{J})=\overset{l}{\underset{h=1}{\sum }}E_{H_{J_{h}}}\{I\{\cup_{j\in J_{h}}[P_{j,1}\leq w_{j,J}c_{J,1}]\;\text{or}\;[P_{j,2}\leq w_{j,J}c_{J,2}]\}$$
  so that its conditional expectation is
$$\begin{array}{ll} E_{H_{J}}(\phi_{J}|\chi_{1}) & =\overset{l}{\underset{h=1}{\sum }}E_{H_{J_{h}}}\left\{I\left[\cup_{j\in J_{h}}(P_{j,2}\leq w_{j,J}c_{J,2})|(p_{j,1},j\in J_{h})\right]\right\}\\ & =B_{J}(\chi_{1}) \end{array}$$
  It follows that
$$\begin{array}{ll} E_{H_{J}}({\tilde{\varphi }}_{J}) & =E_{\chi_{1}}E_{H_{J}}[{\tilde{\varphi }}_{J}|\chi_{1}]\leq E_{\chi_{1}}B_{J}(\chi_{1})=E_{\chi_{1}}E_{H_{J}}[\phi_{J}|\chi_{1}]\\ & =E_{H_{J}}(\phi_{J})=\alpha \end{array}$$
  Since $B_{J}(\chi_{1})$ is the sum of partial conditional error rates of parametric tests, we refer to (26) as the PCER condition for the mixed case. Notice that since $\varphi_{J}\leq \phi_{J}$, the PCER condition is satisfied by the preplanned test Therefore, as with the nonparametric and parametric cases, so also for the mixed case the preplanned testing procedure can be performed if, after examining the stage one data, there are no adaptations.
  Operationally, the PCER condition implies that the stage two critical value ${\tilde{c}}_{J,2}$ for the adapted nonparametric test of $H_{J}$ should satisfy the constraint
(27) $$\overset{l}{\underset{h=1}{\sum }}P_{H_{J_{h}}}\{\cup_{j\in J_{h}}{\tilde{P}}_{j,2}\leq {\tilde{w}}_{j,J}{\tilde{c}}_{J,2})|(p_{j,1},j\in J_{h})\}\leq B_{J}(\chi_{1})$$
  If the $J_{h},h=1,\ldots l,$ are all singletons, condition (27) reduces to (21), the operational PCER condition for the nonparametric case, whereas if l=1, (27) specializes to (24) the operational CER condition for parametric tests.

#### 3.2.3 Final Analysis at the End of Stage Two

For the final analysis at the end of stage two, adapted tests ${\tilde{\varphi }}_{J}$ satisfying the above PCER or CER conditions must be performed for all intersection hypotheses $H_{J},J\in {𝒥}^{+}$. Observe first that for the nonparametric and mixed case tests it is possible to have $B_{J}(\chi_{1})\geq 1$, thereby implying that the PCER condition holds no matter how the test of $H_{J}$ is adapted and regardless of the stage two data. In this case, therefore, one can choose ${\tilde{\varphi }}_{J}=1$ as the adapted test and reject $H_{J}$ at stage one itself. For all other cases it is convenient to partition the set ${𝒥}^{+}$ into three non‐overlapping subsets, ${𝒥}^{+}={𝒥}_{A}\cup {𝒥}_{B}\cup {𝒥}_{C}$, such that, depending on the availability of stage two data, ${\tilde{\varphi }}_{J}$ is defined appropriately within each subset.

 
${𝒥}_{A}=𝒫(I_{2})\cap {𝒥}^{+}:$ Since stage two data will be available for testing the individual hypotheses $\left\{H_{j},j\in I_{2}\right\}$, adapted tests ${\tilde{\varphi }}_{J}$ can be defined for all $J\in {𝒥}_{A}$, as we shall show below.
 
${𝒥}_{B}=𝒫(I_{1}^{*}\setminus I_{2})\cap {𝒥}^{+}:$ Since stage two data will not be available for testing the individual hypotheses $\left\{H_{j},j\in I_{1}^{*}\setminus I_{2}\right\}$ we will set ${\tilde{\varphi }}_{J}=0$ for all $J\in {𝒥}_{B}$.
 
${𝒥}_{C}={𝒥}^{+}\setminus \{{𝒥}_{A}\cup {𝒥}_{B}\}:$ Consider any $J\in {𝒥}_{C}$. Since $J\notin {𝒥}_{A}$ and $J\notin {𝒥}_{B}$, stage two data will be available for testing some individual hypotheses $H_{j},j\in J$ but not for others. In particular stage two data will be available only for testing the individual hypotheses $H_{j},j\in J\cap I_{2}$. Therefore the adapted test ${\tilde{\varphi }}_{J}$ will depend on the stage two data only through the stage two cumulative p values for hypotheses $H_{j}$ with $j\in J\cap I_{2}$.

We now show how, based on the above partitioning, the adapted stage two tests ${\tilde{\varphi }}_{J}$ may be constructed for all $J\in {𝒥}^{+}$ so as to satisfy the PCER condition (21) if nonparametric, the CER condition (24) if parametric, and the PCER condition (27) if mixed, thereby ensuring that in all cases $E_{H_{J}}({\tilde{\varphi }}_{J})\leq \alpha$. In all cases the adapted p value for testing $H_{j}$ at stage two is computed as

(28) $${\tilde{p}}_{j,2}=1-\Phi (\sqrt{{\tilde{t}}_{j}}\;\Phi^{-1}(1-p_{j,1})+\sqrt{1-{\tilde{t}}_{j}}\;\Phi^{-1}(1-{\tilde{p}}_{j,(2)})$$

where ${\tilde{t}}_{j}$, ${\tilde{p}}_{j,(2)}$, and ${\tilde{w}}_{j,J}$ indicate the actual information fraction after sample size reassessment, the incremental stage two p value, and adapted graph‐based weights that may differ due to adaptation of the pre‐specified design at the end of stage one. If there is no adaptation, ${\tilde{p}}_{j,2}$ reverts to (9) the p value for the pre‐planned test at stage two.

 
**Nonparametric** If $J\in {𝒥}_{A}$
(29) $$\begin{array}{l} {\tilde{\varphi }}_{J}=\left\{\begin{array}{ll} 1 & \text{if}\;B_{J}\geq 1\\ 1 & \text{if}\;\cup_{j\in J}\{{\tilde{p}}_{j,2}\leq {\tilde{w}}_{j,J}{\tilde{c}}_{J,2}\}\\ & \;\text{where}\;{\tilde{c}}_{J,2}\;\text{satisfies the PCER condition}\\ & \;\underset{j\in J}{\sum }P_{H_{j}}\{({\tilde{P}}_{j,2}\leq {\tilde{w}}_{j,J}{\tilde{c}}_{J,2})|(p_{j,1},j\in J)\}=B_{J}(\chi_{1})\\ 0 & \text{otherwise} \end{array}\right. \end{array}$$
  If $J\in {𝒥}_{B}$, ${\tilde{\varphi }}_{J}=0$. If $J\in {𝒥}_{C}$, replace J with $J\cap I_{2}$ in the construction of ${\tilde{\varphi }}_{J}$ above, except in the indices of ${\tilde{w}}_{j,J},{\tilde{c}}_{J,2}$ and $B_{J}$, where the index J remains unchanged.
 
**Parametric** If $J\in {𝒥}_{A}$,
(30) $${\tilde{\varphi }}_{J}=\left\{\begin{array}{ll} 1 & \text{if}\;\cup_{j\in J}\{{\tilde{p}}_{j,2}\leq {\tilde{w}}_{j,J}{\tilde{c}}_{J,2}\}\\ & \;\text{where}\;{\tilde{c}}_{J,2}\;\text{satisfies the CER condition}\\ & \;P_{H_{J}}\{[\cup_{j\in J}({\tilde{P}}_{j,2}\leq {\tilde{w}}_{j,J}{\tilde{c}}_{J,2})]|(p_{j,1},j\in J)\}=B_{J}(\chi_{1})\\ 0 & \text{otherwise} \end{array}\right.$$
  If $J\in {𝒥}_{B}$, ${\tilde{\varphi }}_{J}=0$. If $J\in {𝒥}_{C}$, replace J with $J\cap I_{2}$ in the construction of ${\tilde{\varphi }}_{J}$ above, except in the indices of ${\tilde{w}}_{j,J},{\tilde{c}}_{J,2}$ and $B_{J}$, where the index J remains unchanged.
 
**Mixed** We use the partition of the index set J defined in (16). Then, if $J\in {𝒥}_{A}$, the mixed parametric test of $H_{J}$ is given by
(31) $${\tilde{\varphi }}_{J}=\left\{\begin{array}{ll} 1\; & \text{if}\;B_{J}\geq 1,\\ 1\; & \text{if}\;\cup_{h=1}^{l}\cup_{j\in J_{h}}\{{\tilde{p}}_{j,2}\leq {\tilde{w}}_{j,J}{\tilde{c}}_{J,2}\},\\ \; & \text{where}\;{\tilde{c}}_{J,2}\;\text{satisfies the PCER condition}\\ \; & \overset{l}{\underset{h=1}{\sum }}P_{H_{J_{h}}}\{[\cup_{j\in J_{h}}({\tilde{P}}_{j,2}\leq {\tilde{w}}_{j,J}{\tilde{c}}_{J,2})]|(p_{j,1},j\in J_{h})\}\\ \; & \;=B_{J}(\chi_{1}),\\ 0\; & \text{otherwise} \end{array}\right.$$
  If $J\in {𝒥}_{B}$, ${\tilde{\varphi }}_{J}=0$. If $J\in {𝒥}_{C}$, replace $J_{h}$ with $J_{h}\cap I_{2}$ in the construction of ${\tilde{\varphi }}_{J}$ above, except in the indices of ${\tilde{w}}_{j,J},{\tilde{c}}_{J,2}$ and $B_{J}$, where the index J remains unchanged. The mixed case test (31) is the most general way to test any adapted intersection hypothesis $H_{J}$. It specializes to the nonparametric test (29) if the $\left\{J_{h},h=1,2,\ldots l\right\}$, are all singletons, and specializes to the parametric test (30) if l=1.

Supplementary Remarks

1. In all three cases above, it is assumed that if $J\in {𝒥}_{C}$ no weight is given to dropped hypothesis, such that $\sum_{j\in J\cap I_{2}}{\tilde{w}}_{j,J}=1$, and ${\tilde{w}}_{j,J}=0$ for all $j\in J\setminus I_{2}$.
2. For many settings, the conditional probabilities (21), (24) and (27) can be computed from multivariate normal distributions for z‐statistics obtained by inverse normal transformations of corresponding p value statistics.

#### 3.2.4 Illustrative Example of the Conditional Error Rate Method

We will repeat the analysis of the schizophrenia trial at one‐sided α=0.025, this time by the CER method, keeping the weighting strategy and all other design parameters the same as in Section 3.1.3. The first step is to prespecify the tests for all the intersection hypotheses $H_{J},J\subseteq I_{1}$ that would be performed if there were no adaptation at the end of stage one. For nonparametric tests (12) this involves specifying the critical cutoff values $w_{j,J}c_{J,i}$, for corresponding elementary p values $p_{j,i},j\in J,i=1,2$. We illustrate below with a couple examples.

Consider prespecification of the test of $H_{J}$ where J={1,2,3,4}. From Table 3, $w_{1,J}=0.5,w_{2,J}=0.5,w_{3,J}=0,w_{4,J}=0$. Therefore, the elementary p values $p_{3,i}$ and $p_{4,i}$, having zero weights associated with them, will play no role in the test of $H_{J}$. Acceptance or rejection of $H_{J}$ will depend solely on $p_{1,i}$ and $p_{2,i}$, i=1,2. Since $\text{corr}(Z_{P_{1,i}},Z_{P_{2,i}})=0.5$, we are in the parametric setting and need to evaluate $c_{J,1}$ from (13) and $c_{J,2}$ from (14). We will be using the [32] error spending function (8) and taking the interim look at information fraction t=0.5. Thus

(32) $$\alpha_{J,1}=2-2\Phi \left(\frac{z_{\alpha /2}}{\sqrt{t}}\right)$$

Solving (13) for the critical constant $c_{J,1}$ we have,

$$P_{J}\{\cup_{j\in \{1,2\}}[P_{j,1}\leq w_{j,J}c_{J,1}]\}=0.001525$$

whereupon $c_{J,1}=0.001564$. Similarly, solving (14) for the critical constant $c_{J,2}$ we have

$$\begin{array}{ll} & 0.001525+P_{H_{J}}\{\cap_{j\in J}[P_{j,1}>0.001564w_{j,J}]\;\text{and}\\ & \;\cup_{j\in J}[P_{j,2}\leq w_{j,J}c_{J,2}]\}=\alpha \end{array}$$

whereupon $c_{J,2}=0.02633$. Thus, the prespecified p value boundaries for $\varphi_{J}$ in (15) are $w_{1,J}c_{J,1}=w_{2,J}c_{J,1}=0.000782$, $w_{3,J}c_{J,1}=w_{4,J}c_{J,1}=0$ at stage one, and $w_{1,J}c_{J,2}=w_{2,J}c_{J,2}=0.0132,w_{3,J}c_{J,2}=w_{4,J}c_{J,2}=0$ at stage two.

Next consider the prespecification of the test of $H_{J}$ where J={2,3,4}. From Table 3, $w_{2,J}=0.75,w_{3,J}=0.25,w_{4,J}=0$. Since $w_{4,J}=0$, the elementary p values $p_{4,i},i=1,2,$ will play no role in the test of $H_{J}$ and we need only concern ourselves with critical cut‐off values for $p_{2,i}$ and $p_{3,i}$. Since the correlation between $P_{2,i}$ and $P_{3,i}$ is unknown, we are in the nonparametric setting. Solving the group sequential Equation (10) for $c_{J,1}$ yields $c_{J,1}=\alpha_{J,1}=0.001525$. Therefore the prespecified stage one p value boundaries for $\varphi_{J}$ in (12) are $w_{2,J}c_{J,1}=0.001144,w_{3,J}c_{J,1}=0.000381,w_{4,J}c_{J,1}=0$. Solving the group sequential equation (11) for $c_{J,2}$ yields $c_{J,2}=0.024409$. Thus, the corresponding pre‐specified stage two p value boundaries in (12) are $w_{2,J}c_{J,2}=0.0183,w_{3,J}c_{J,2}=0.00610,w_{4,J}c_{J,2}=0$.

The weights $\left\{w_{j,J},j\in J\right\}$ and the prespecified stage one and stage two p value boundaries are displayed in Table 8 for all intersection hypotheses $H_{J},J\subseteq I_{1}$.

**TABLE 8:** Prespecified Stage One and Stage Two Boundaries for CER Method.

Intersection hypotheses $\left(H_{J}\right)$	Weights $\left\{w_{j,J},j\in J\right\}$	P value boundaries		Type of test
		Stage one $\left(w_{j,J}c_{J,1}\right)$	Stage two $\left(w_{j,J}c_{J,2}\right)$	
$H_{1}\cap H_{2}\cap H_{3}\cap H_{4}$	{0.5, 0.5, 0, 0}	{0.000782, 0.000782, 0, 0 }	{0.0132, 0.0132, 0, 0}	Parametric
$H_{2}\cap H_{3}\cap H_{4}$	{0.75, 0.25, 0}	{0.00114, 0.000381, 0}	{0.0183, 0.00610, 0}	Nonparametric
$H_{1}\cap H_{3}\cap H_{4}$	{0.75, 0, 0.25}	{0.00114, 0, 0.000381}	{0.0183, 0, 0.00610}	Nonparametric
$H_{1}\cap H_{2}\cap H_{4}$	{0.5, 0.5, 0}	{0.000782, 0.000782, 0}	{0.0132, 0.0132, 0}	Parametric
$H_{1}\cap H_{2}\cap H_{3}$	{0.5, 0.5, 0}	{0.000782, 0.000782, 0}	{0.0132, 0.0132, 0}	Parametric
$H_{3}\cap H_{4}$	{0.5, 0.5}	{0.000782, 0.000782}	{0.0132, 0.0132}	Parametric
$H_{2}\cap H_{4}$	{1, 0}	{0.001525, 0}	{0.0245, 0}	NA
$H_{2}\cap H_{3}$	{0.75, 0.25}	{0.00114, 0.000381}	{0.0183, 0.00610}	Nonparametric
$H_{1}\cap H_{4}$	{0.75, 0.25}	{0.00114, 0.000381}	{0.0183, 0.00610}	Nonparametric
$H_{1}\cap H_{3}$	{1, 0}	{0.001525, 0}	{0.0245, 0}	NA
$H_{1}\cap H_{2}$	{0.5, 0.5}	{0.000782, 0.000782}	{0.0132, 0.0132}	Parametric
$H_{4}$	NA	0.001525	0.0245	NA
$H_{3}$	NA	0.001525	0.0245	NA
$H_{2}$	NA	0.001525	0.0245	NA
$H_{1}$	NA	0.001525	0.0245	NA

Suppose the stage one p values for the elementary hypotheses in $I_{1}$ are $p_{1.1}=0.00045$, $p_{2,1}=0.0952$, $p_{3,1}=0.0225$, and $p_{4,1}=0.1104$. Applying these observations to the stage one p value boundaries in Table 8, it is seen that every intersection hypothesis containing $H_{1}$ is rejected, whereas all other intersection hypotheses are retained. Therefore, $H_{1}$ is rejected under closed testing. Formally, the index set ${𝒥}^{r}=\{(1,2,3,4),(1,3,4),(1,2,4),(1,2,3),(1,4),(1,3),(1,2),(1)\},I_{r1}=\{1\}$, $I_{1}^{*}=\{2,3,4\}$, and ${𝒥}^{+}=\{(2,3,4),(3,4),(2,4),(2,3),(4),(3),(2)\}.$

In order to complete a level‐α closed test for all the elementary hypotheses $H_{j},j\in I_{1}$, we are required to test all the intersection hypotheses $H_{J},J\in {𝒥}^{+}$ with local level‐α tests at the end of stage two. Before proceeding to stage two, however, there is the option to make adaptive changes to the on going trial. We have indicated in Section 3.2 several ways in which the trial may be adapted including dropping of hypotheses, sample size re‐estimation and altering the testing strategy. Should any of these changes be implemented it will be necessary to replace the pre‐specified tests $\varphi_{J}$ with modified tests ${\tilde{\varphi }}_{J}$ for all $J\in {𝒥}^{+}$ such that the modified tests satisfy the required CER or PCER conditions. Accordingly, Table 9 displays $B_{J}(\chi_{1})$ values as computed by (19) for the nonparametric tests and by (22) for the parametric tests, for all the intersection hypotheses $H_{J},J\in {𝒥}^{+}$.

**TABLE 9:** Required Values of $B_{J}(\chi_{1})$ for all $J\in {𝒥}^{+}$ conditional on stage one p values.

Intersection hypotheses ($J\in {𝒥}^{+}$)	Weights $\left\{w_{j,J},j\in J\right\}$	$B_{J}(\chi_{1})$
$H_{2}\cap H_{3}\cap H_{4}$	{0.75, 0.25, 0}	0.1117
$H_{3}\cap H_{4}$	{0.5, 0.5}	0.1415
$H_{2}\cap H_{4}$	{1, 0}	0.0702
$H_{2}\cap H_{3}$	{0.75, 0.25}	0.1117
$H_{4}$	1	0.0594
$H_{3}$	1	0.2179
$H_{2}$	1	0.0702

We illustrate below how the conditional probabilities comprising each $B_{J}(\chi_{1})$ value in Table 9 are calculated for the case J={3,4}. From Table 8, the stage two p value boundaries for the intersection hypothesis $H_{3}\cap H_{4}$ are $w_{J,3}c_{J,2}=w_{J,4}c_{J,2}=0.01317$. Thus, by (22),

(33) $$\begin{array}{ll} B_{J}(\chi_{1}) & =P_{H_{J}}\left\{\cup_{j=3}^{4}(P_{j,2}\leq 0.01317|p_{3,1}=0.0225,p_{4,1}=0.1104)\right\} \end{array}$$

Upon substituting the expression for $P_{j,2}$ given by (9) into Equation (33) we have

(34) $$\begin{array}{ll} B_{J}(\chi_{1}) & =P_{H_{J}}\{(Z_{3,(2)}\geq t^{-0.5}z_{0.01317}-z_{0.0225})\\ & \;\cup (Z_{4,(2)}\geq t^{-0.5}z_{0.01317}-z_{0.1104})\} \end{array}$$

where $Z_{j,(2)}=\Phi^{-1}(1-P_{j,(2)})$, j=3,4, are standard normal random variables with $\text{cov}(Z_{3,(2)},Z_{4,(2)})=0.5$, and t=0.5. We evaluate the right hand side of (34) by bivariate normal integration to obtain $B_{J}(\chi_{1})=0.1415$.

Suppose it is decided to drop $H_{3}$, the secondary endpoint for the high dose arm, on the grounds that, having already rejected $H_{1}$, the primary hypothesis for the high dose arm, it is preferable to now focus all remaining sample size resources only on the low dose arm. By this decision, the additional 35 subjects who would have been randomized to the high dose arm at stage two will now be allocated to the low dose or control arms, leading to a second stage incremental sample size of 52 and 53 in the low dose and control group, respectively. The adapted information fraction is thus

$$\tilde{t}=\frac{{\left[1/35+1/35\right]}^{-1}}{\left({\left[1/35+1/35\right]}^{-1}+{\left[1/52+1/53\right]}^{-1}\right)}=0.4$$

**FIGURE 3:**
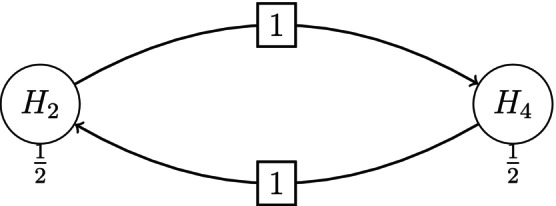
Graph of Stage Two Testing Strategy for Schizophrenia Trial using CER Method.

We are now left with only the two elementary hypotheses $H_{2}$ and $H_{4}$, indexed by $I_{2}=\{2,4\}$. Suppose that it is also decided to alter the testing strategy and treat the two endpoints for the low dose arm as co‐primaries, and to therefore assign equal weight to $H_{2}$ and $H_{4}$. The graph representing the revised stage two testing strategy is displayed in Figure 3.

Because of the above adaptive changes at the end of stage one, the p value boundaries at stage two for the adapted tests ${\tilde{\varphi }}_{J},J\in {𝒥}^{+}$ must be recomputed. For this purpose, we first partition ${𝒥}^{+}$ into the three nonoverlapping subsets ${𝒥}_{A}=\{(2),(4),(2,4)\}$, ${𝒥}_{B}=\{(3)\}$ and ${𝒥}_{C}=\{(2,3,4),(3,4),(2,3)\}$ as defined in Section 3.2.3. Then ${\tilde{\varphi }}_{\left\{3\right\}}=0$. The boundaries for the remaining hypotheses are displayed in Table 10 for all $J\in {𝒥}_{A}$ and in Table 11 for all $J\in {𝒥}_{C}$. The Table 10 boundaries were evaluated in accordance with Equation (21). The Table 11 boundaries were evaluated in accordance with Equation (21) after replacing each index set J with $J\cap I_{2}$ in the summation, except in the indices of ${\tilde{w}}_{j,J},{\tilde{c}}_{J,2}$ and $B_{J}$.

**TABLE 10:** Recomputed stage two p value boundaries for intersection hypotheses $H_{J},J\in {𝒥}_{A}$.

Index set J	Weights $\left\{{\tilde{w}}_{j,J},j\in J\right\}$	Boundary type	Boundary values
{2, 4}	{0.5, 0.5}	Nonparam	${\tilde{p}}_{2,2}\leq 0.0137\;\text{or}\;{\tilde{p}}_{4,2}\leq 0.0137$
{4}	1	NA	${\tilde{p}}_{4,2}\leq 0.0237$
{2}	1	NA	${\tilde{p}}_{2,2}\leq 0.0244$

**TABLE 11:** Recomputed stage two p value boundaries for intersection hypotheses $H_{J},J\in {𝒥}_{C}$.

Index set J	Restricted set $J\cap I_{2}$	Weights $\left\{{\tilde{w}}_{j,J\cap I_{2}},j\in J\cap I_{2}\right\}$	Boundary type	Boundary values
{2, 3, 4}	{2, 4}	{0.5, 0.5}	Nonparam	${\tilde{p}}_{2,2}\leq 0.0210\;\text{or}\;{\tilde{p}}_{4,2}\leq 0.0210$
{3,4}	{4}	1	NA	${\tilde{p}}_{4,2}\leq 0.0541$
{2,3}	{2}	1	NA	${\tilde{p}}_{2,2}\leq 0.0382$

The trial proceeds to stage two. Suppose that at the end of stage two the incremental p values are ${\tilde{p}}_{2,(2)}=0.0299,{\tilde{p}}_{4,(2)}=0.0586$ leading, in accordance with (28), to cumulative p values over the two stages of the trial of ${\tilde{p}}_{2,2}=0.0111$ and ${\tilde{p}}_{4,2}=0.0234$. Then it is seen from Tables 10 and 11 that all the p value boundaries are crossed. Therefore $H_{2}$ and $H_{4}$ are both rejected under closed testing.

Suppose we decide not to drop $H_{3}$ at stage two, but instead decide to change the stage two weights of the hypotheses in $I_{2}$ from $w_{2,I_{2}}=0.75$, $w_{3,I_{2}}=0.25$, $w_{4,I_{2}}=0$, to ${\tilde{w}}_{2,I_{2}}=0.5$, ${\tilde{w}}_{3,I_{2}}=0.25$, ${\tilde{w}}_{4,I_{2}}=0.25$. As a result of this adaptation, the stage two test of the intersection hypothesis $H_{2}\cap H_{3}\cap H_{4}$ becomes a mixed test in which the index set $I_{2}=\{2,3,4\}$ is partitioned into the union of $J_{1}=\{2\}$ and $J_{2}=\{3,4\}$. We now illustrate how this mixed‐case intersection hypothesis may be tested. Following Equation (31), the adapted critical cut‐off ${\tilde{c}}_{I_{2},2}$ satisfies

(35) $$\begin{array}{ll} & P_{H_{2}}\{{\tilde{P}}_{2,2}\leq {\tilde{w}}_{2,I_{2}}{\tilde{c}}_{I_{2},2}|p_{2,1}=0.0952\}\\ & \;+P_{H_{\left\{3,4\right\}}}\{({\tilde{P}}_{3,2}\leq {\tilde{w}}_{3,I_{2}}{\tilde{c}}_{I_{2},2})\cup ({\tilde{P}}_{4,2}\leq w_{4,I_{2}}{\tilde{c}}_{I_{2},2})|p_{3,1}\\ & \;=0.0225,p_{4,1}=0.1104\}=0.1117 \end{array}$$

Since the stage two statistics are specified by (9) and t=0.5, we can rewrite (35) as

(36) $$\begin{array}{ll} & P_{H_{2}}(Z_{2,(2)}\geq \sqrt{2}z_{q_{2}}-z_{0.0952})\\ & \;+P_{H_{\left(3,4\right)}}\left\{\left(Z_{3,(2)}\geq \sqrt{2}z_{q_{3}}-z_{0.0225})\cup (Z_{4,(2)}\geq \sqrt{2}z_{q_{4}}-z_{0.1104}\right)\right\}\\ & \;=0.1117 \end{array}$$

where $q_{j}=w_{j,I_{2}}c_{I_{2},2}$, and $z_{\gamma }=\Phi^{-1}(1-\gamma )$. Using the R function pmvnorm belonging to the library mvtnorm, to search for the value of ${\tilde{c}}_{I_{2},2}$ that satisfies Equation (36) we obtain ${\tilde{c}}_{I_{2},2}=0.027492$. Therefore by (31), the intersection hypothesis $H_{2}\cap H_{3}\cap H_{4}$ is rejected if either ${\tilde{p}}_{2,2}\leq {\tilde{w}}_{2,I_{2}}{\tilde{c}}_{I_{2,2}}=0.0137$, or ${\tilde{p}}_{3,2}\leq {\tilde{w}}_{3,I_{2}}{\tilde{c}}_{I_{2},2}=0.006873$, or ${\tilde{p}}_{4,2}\leq {\tilde{w}}_{4,I_{2}}{\tilde{c}}_{I_{2},2}=0.006873$.

## 4 Simulation study

**FIGURE 4:**
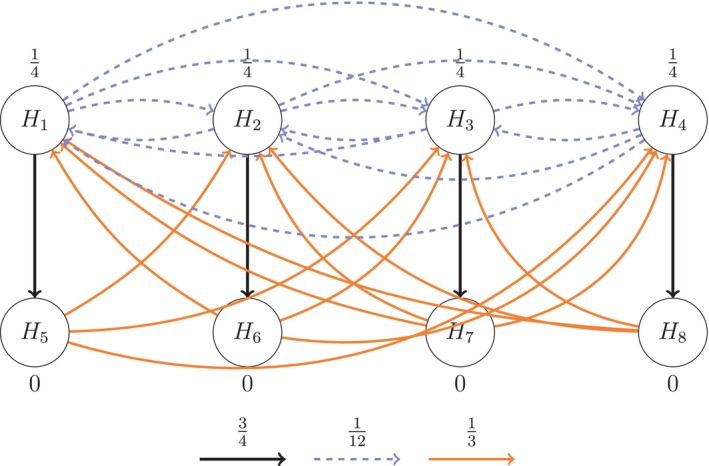
Graphical representation of design for comparing PVcombo and CER in a trial with four treatment arms and two endpoints. The black arrow indicates the proportion of weight that is reallocated from the primary to the respective secondary hypotheses once the primary hypothesis is rejected. The blue dashed arrows indicate that the remaining weight is re‐allocated to the other primary hypotheses. The orange arrows define that after the rejection of a secondary hypothesis, its weight is reallocated to the other primary hypotheses.

In order to compare the operating characteristics of the P value combination (PVCombo) and partial conditional error rate (CER) methods, a simulation study was conducted. We generalize the graph‐based test in Figure 1 for a trial with four treatment arms that were compared in a pair‐wise manner to a common control arm. Each treatment versus control comparison included two hypotheses, one primary and one secondary. A closed test of the eight hypotheses was conducted over two stages with early rejection of efficacious hypotheses at the end of stage one based on the [32] error spending function (8) with t=0.5 and γ=0.025. A graph‐based weighting strategy was adopted for testing all the intersection hypotheses of the closed test. The testing of intersection hypotheses exploited known correlations among p values with parametric tests and reverted to Bonferroni‐based testing when the correlations were unknown, as described in Sections 3.1 and 3.2. The corresponding graph is depicted in Figure 4, where $H_{1},\ldots ,H_{4}$ are the null hypotheses of no treatment effect for the four primary endpoints and $H_{5},\ldots ,H_{8}$ are the null hypotheses of no treatment effect for the corresponding secondary endpoints. Initially, all the weight is distributed equally among the primary hypotheses. If a primary hypothesis is rejected, 3/4 of its weight is transferred to the corresponding secondary endpoint. The remaining weight is equally distributed among the other primary hypotheses. If a secondary endpoint is rejected, its weight is transferred evenly to the remaining primary hypotheses.

We assume that the endpoints are normally distributed and we aim to test the null hypotheses that the difference in means in each endpoint between each treatment and control group are equal. The marginal p values utilized in the hypothesis tests of each simulation were obtained from one‐sided t‐tests with degrees of freedom under the assumption of equal variance. The nominal familywise error rate was set to 2.5%. Let $\delta_{j},j=1,\ldots ,4$ be the effect size of treatment j versus control for the primary endpoint. For this investigation we assumed that the effect size of the secondary endpoint equals that of the corresponding primary endpoint. That is, $\delta_{j}=\delta_{j+4},j=1,\ldots 4$. Additionally we assumed that there is a common standard deviation σ=1 for all treatment and control arms, for both endpoints.

**FIGURE 5:**
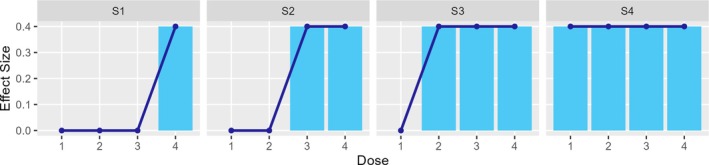
Graphical representation of the effect size scenarios considered in the simulation study.

We considered trials with a preplanned number of 100 subjects per arm and an interim analysis at 50% of the enrollment for early rejection of statistically significant hypotheses and adaptive changes to the design. In the power simulations we assumed a correlation of 0.5 between the observations in the primary and secondary endpoint. To assess the type 1 error rate we additionally considered the case of no correlation and a correlation of 0.8. The simulations compared the disjunctive and conjunctive powers of the two methods over four alternative hypothesis scenarios and four decision rules for treatment selection at the end of stage one. The four alternative hypothesis scenarios, labelled S1 to S4 respectively, represent, one, two, three or four active treatment arms, with activity being defined as $\delta_{j}=0.4$, as shown in Figure 5. For each scenario, we conducted 500,000 simulated trials for the Combo Method. For the CER Method, we conducted 100,000 stage one simulations and, for each stage one result, we conducted 100 stage two simulations, and then aggregated the outcomes. As, for the CER method, the main computational effort is the computation of adjusted boundaries based on the first stage outcome, this approach is more efficient than a classical Monte Carlo simulation, simulating whole trials.

Four decision rules were utilized for dropping ineffective treatment arms at the end of stage one, based on marginal p values. They ranged from extreme conservatism to extreme aggression for dropping treatment arms and are thus titled as conservative, normal, aggressive, and ultra aggressive. These rules are tabulated in Table 12. If a treatment arm was dropped at the end of stage one, the remaining subjects were reassigned to the arms going forward to stage two.

**TABLE 12:** Decision rules for dropping treatment arms.

Decision rule	Treatment dropping criterion
Conservative	Drop if p≥0.75
Normal	Drop if p≥0.5
Aggressive	Drop if p≥0.25
Ultra aggressive	Select only the treatment with the smallest p value

**FIGURE 6:**
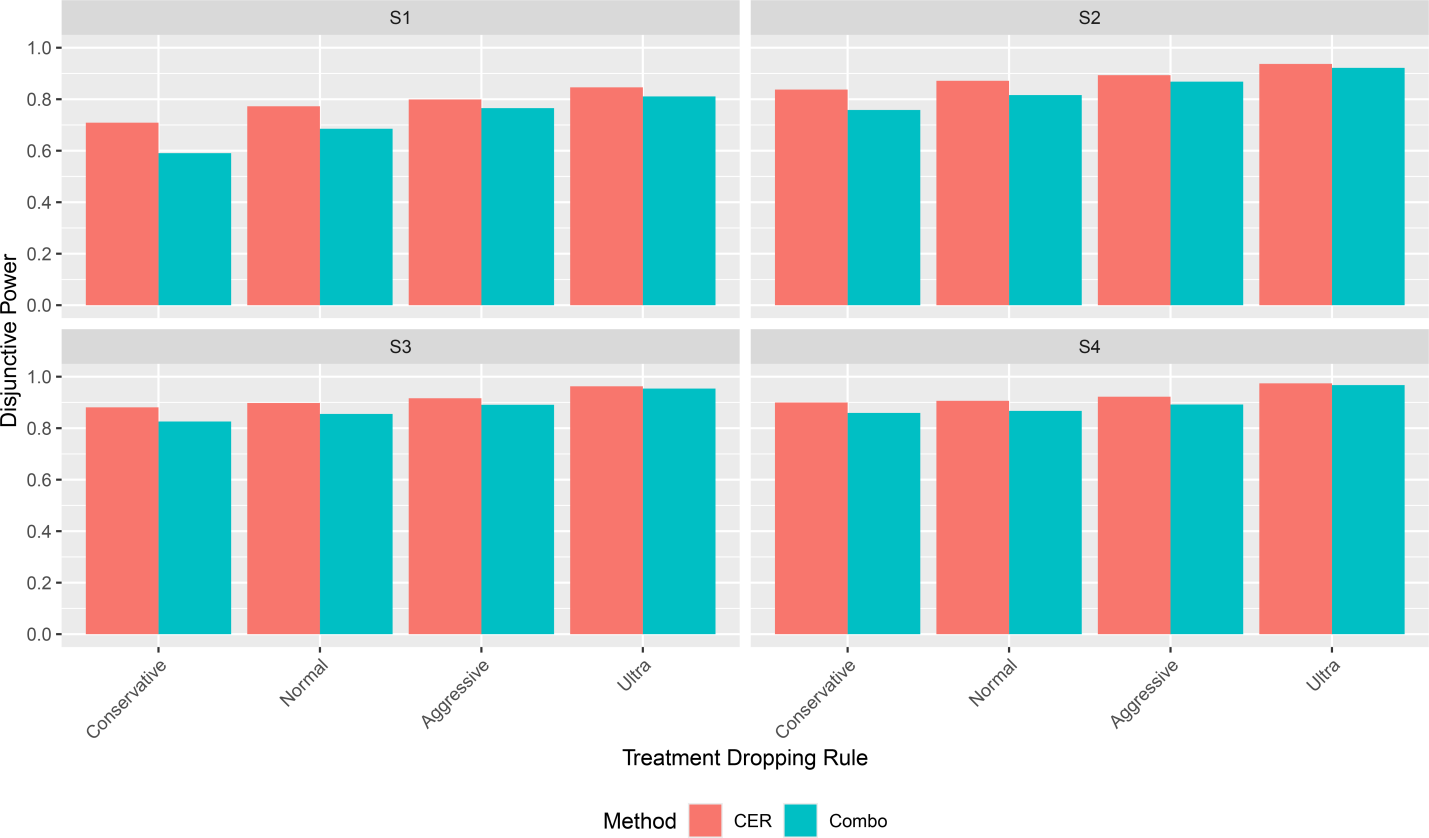
Disjunctive power of CER vs Combo by treatment dropping rule and scenario.

**FIGURE 7:**
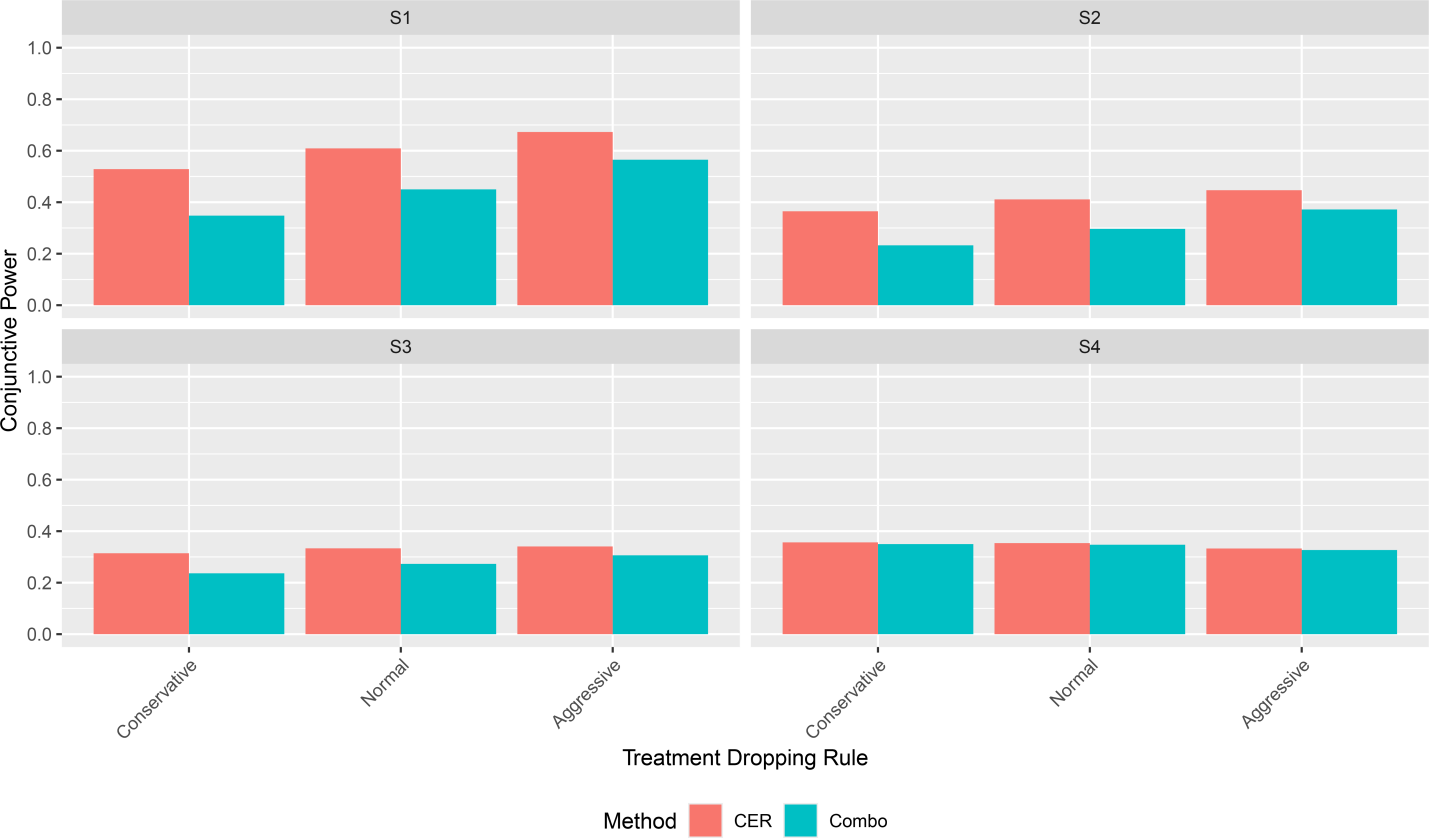
Conjunctive power of the CER vs Combo method by treatment dropping rule and scenario. For the Ultra Aggressive rule, that selects only a single arm for the second stage, no conjunctive power is reported.

Figure 6 compares the disjunctive power for the CER and Combo methods in four panels, one for each alternative hypothesis scenario. Within each panel, the comparisons are by treatment dropping decision rule. Figure 7 does the same for conjunctive power. Numeric simulation results are given in Table 13.

**TABLE 13:** Disjunctive power, conjunctive power and FVER for the conditional error rate (CER) and the combination test (Comb) methods under scenarios S1, S2, S3, S4 and different rules to drop doses.

Scenario	Dropping rule	Method	Disjunctive (%)	Conjunctive (%)	FVER (%)
S1	Conservative	CER	70.9	52.8	2.38
		Combo	59.0	34.8	1.34
	Moderate	CER	77.3	60.9	2.39
		Combo	68.5	45.0	1.47
	Aggressive	CER	79.9	67.3	2.33
		Combo	76.5	56.5	1.73
	Ultra aggressive	CER	84.6		0.63
		Combo	81.1		0.66
S2	Conservative	CER	83.7	36.5	2.26
	Combo	75.8	23.2	1.62
	Moderate	CER	87.1	41.1	2.26
		Combo	81.6	29.7	1.73
	Aggressive	CER	89.3	44.7	2.17
		Combo	86.8	37.2	1.87
	Ultra aggressive	CER	93.7		0.27
		Combo	92.2		0.28
S3	Conservative	CER	88.1	31.4	2.06
	Combo	82.6	23.6	2.04
	Moderate	CER	89.8	33.3	2.04
		Combo	85.5	27.3	2.01
	Aggressive	CER	91.6	34.1	1.88
		Combo	89.1	30.6	1.85
	Ultra aggressive	CER	96.3		0.13
		Combo	95.4		0.13
S4	Conservative	CER	90.0	35.6	
	Combo	85.9	35.0	
	Moderate	CER	90.6	35.4	
		Combo	86.7	34.7	
	Aggressive	CER	92.2	33.3	
		Combo	89.2	32.7	
	Ultra aggressive	CER	97.4		
		Combo	96.7		

*Note:* For the ultra aggressive rule no conjunctive power is given. The standard errors for the power estimates are all below 0.1%. The standard errors for the FVER estimates are all below 0.022%. In scenario S4 all null hypotheses are false and no type 1 error can occur.

The following consistent pattern emerges from an examination of these figures and table.

1. It is seen from Figure 6 that the CER method has greater disjunctive power than the Combo in all considered scenarios.
2. The gain in disjunctive power of CER over Combo within each alternative hypothesis scenario is greatest for the conservative treatment dropping rule and diminishes with increasing aggressiveness for dropping losers.
3. The power gains of the CER method over Combo can be surprisingly large. For the conservative treatment dropping rule the gain in disjunctive power ranges from 4% to 12%, while for the normal rule the range is 4% to 9%.
4. Within each panel of Figure 6, it is seen that the absolute disjunctive power for both methods increases with increasing aggressiveness of dropping treatments.
5. From a visual inspection of Figure 6, it seems to be the case that the power gain of CER over Combo diminishes as the absolute power of both methods increases. This is to be expected since there is an upper bound on power. It does imply, however, that the settings in which the power gain is higher are precisely those in which it is most needed.
6. We also compared the two methods with respect to conjunctive power (see Figure 7 and Table 13). Again, in all considered scenarios the CER has a higher power with the largest differences with the conservative rule and when only few treatments are effective.

Finally, in order to verify FWER control, we conducted simulations under the global null hypotheses for all four decision rules under the assumption of no correlation, a correlation of 0.5, and a strong correlation of 0.8 among the primary and secondary endpoints (Table 14). The FWER for both methods is controlled at the nominal level within the Monte Carlo error. However, in the considered scenarios, the Combo method is substantially more conservative, with the exception of the ultra aggressive dropping rule. In addition, the FWER does not vary strongly between different strengths of correlation between the primary and secondary endpoint.

**TABLE 14:** The estimated FWER of the two methods under the global null hypothesis. The standard error of the estimates is below 0.022 percentage points for the CER method and below 0.05 percentage points for the COMB method.

		FWER (%) by decision rules			
Correlation	Method	Conservative	Normal	Aggressive	Ultra
0.0	CER	2.48	2.46	2.40	2.18
	Combo	1.14	1.23	1.53	2.34
0.5	CER	2.49	2.47	2.40	2.25
	Combo	1.19	1.29	1.57	2.33
0.8	CER	2.49	2.47	2.40	2.31
	Combo	1.25	1.35	1.62	2.33

## 5 Discussion

This study has advanced the development of adaptive trial design in two ways. First, we have shown how to combine the graph‐based methodology for testing multiple hypotheses with the group sequential methodology for early rejection of hypotheses. Second, we have demonstrated through a simulation study that the CER method for preserving the FWER of complex adaptive designs has greater power than the P value combination method. This reinforces similar findings for the simpler multiarm multi‐stage single endpoint setting [13, 15] and for enrichment designs [34]. The large gains in power with CER compared to Combo under some scenarios and treatment dropping rules suggest that this method should be the preferred option for future trial design. We caution, however, that pre‐trial simulations of all plausible scenarios and decision rules should always be conducted prior to settling on the choice of design. This includes simulations to verify strong control of the FWER.

One reason for the greater power of the CER over the Combo method is the lack of consonance of the closed test (see [30]). For consonant tests, rejection of the global intersection hypothesis implies rejection of at least one individual hypothesis. This does not necessarily hold for non‐consonant tests. Therefore, non‐consonant closed testing procedures are strictly conservative, as rejection of the intersection hypothesis at level α does not imply rejection of an individual hypothesis in the closed test. This also results in a loss of power. The pre‐planned test of the CER method, defined by the graph in Figure 4, is consonant. Therefore, whenever the trial continues with all treatment arms to the second stage, for the CER method a rejection of the intersection hypothesis implies rejection of at least one individual hypothesis in the closed test. However, if treatment arms are dropped at the interim analysis, consonance is lost. This can be seen from Table 14 that shows that for the CER method the FWER decreases for increasingly aggressive treatment dropping rules. For the Combo method in contrast, even if no treatments are dropped, the combination test is not consonant. Here, non‐consonant test decisions are less frequent for more aggressive treatment dropping rules and therefore we also observe a better exhaustion of the significance level as the aggressiveness for dropping doses increases. Overall, although for both CER and the Combo the adaptive test is in general not consonant, the non‐consonance can become much more severe for the Combo than the CER test. While the estimated FWER for the CER test is larger than 2.18% in all considered scenarios, it can fall below 1.2% for the Combo test (Table 14). We have included a PDF file (appendix_consonance.pdf) in the Supporting Information section on the Journal's site with simple numerical examples of graph‐based tests that start out consonant at stage one and lose this property at stage two and also graph‐based tests that start out non‐consonant at stage one and acquire consonance at stage two. Another reason for the loss in power of the combination test is that, in some scenarios, the intersection hypothesis tests using the Combo method have substantially lower power than those using the CER method. This occurs, for example, in scenario S1, where only one arm is effective (data not shown).

While some statistical tests can be uniformly improved by making them consonant (see, e.g., [35]), such improvements are not applicable to adaptive tests unless the adaptation rule is fixed in advance.

We also noted in Section 4 that, for both methods, in the considered scenarios the absolute magnitude of the disjunctive power is lowest for the Conservative drop‐the‐loser rule and increases with increasing aggressiveness for dropping losers. This is readily explained by the fact that if a treatment arm is dropped at the interim analysis time point, the remaining sample size that was committed to that treatment arm at the design stage is re‐allocated to the remaining arms that will be proceeding to stage two.

Although our worked example focused on comparing multiple treatments with multiple endpoints, the framework of graph‐based multiple testing can be applied more generally. Thus, the multiple hypotheses could represent a population of interest and its underlying subgroups, possibly identified by biomarkers. In this setting, the methodology could be used for adaptive population designs. We will develop an application of this type in a subsequent paper.

The testing procedures considered are based on stagewise p values computed from the respective stagewise samples. These may originate from any hypothesis test, provided that, under the respective (intersection) null hypothesis, the distribution of the first‐stage p values is stochastically larger than or equal to the uniform distribution, and the distribution of the second‐stage p values, conditional on the interim data, is also stochastically larger than or equal to the uniform distribution [36, 37]. These conditions are typically satisfied if a conservative test is applied at each stage and the observations at the two stages are independent. However, in settings with delayed or time‐to‐event endpoints, where, at the time of the interim analysis, partial data may be available for some recruited patients but their primary endpoint has not yet been observed, special care is required and additional considerations apply (see, e.g., [38, 39]). Furthermore, for parametric tests, it is additionally required that the p values are based on multivariate normal test statistics. This condition is satisfied by many test procedures, at least asymptotically.

We have confined our investigation to two‐stage designs. The generalization to more than two stages is straightforward for the Combo method, since the same basic algorithm for combining incremental adjusted p values with prespecified weights can be applied. The generalization of the CER method, however, may require more care and is currently a topic for investigation.

AdaptGMCP, an R‐package implementing the methods in this paper, is available in the Supporting Information section on the Journal site along with a PDF file (Illustrative Examples in sections 3.1.3 and 3.2.4.pdf) that demonstrates its application to the worked examples in sections 3.1.3 and 3.2.4 of the paper. AdaptGMCP can also be downloaded from https://github.com/Cytel‐Inc/AdaptiveGMCP.

## Conflicts of Interest

The authors declare no conflicts of interest.

## Supporting information




**Data S1.** Supporting Information.

## Data Availability

The authors have nothing to report.

## Appendix A

The graph used in the simulation study in Section 4 is defined by the initial weight vector (1/4,1/4,1/4,1/4,0,0,0,0) and the transition matrix

$$G=\left[\begin{array}{cccccccc} 0 & \frac{1}{12} & \frac{1}{12} & \frac{1}{12} & \frac{3}{4} & 0 & 0 & 0\\ \frac{1}{12} & 0 & \frac{1}{12} & \frac{1}{12} & 0 & \frac{3}{4} & 0 & 0\\ \frac{1}{12} & \frac{1}{12} & 0 & \frac{1}{12} & 0 & 0 & \frac{3}{4} & 0\\ \frac{1}{12} & \frac{1}{12} & \frac{1}{12} & 0 & 0 & 0 & 0 & \frac{3}{4}\\ 0 & \frac{1}{3} & \frac{1}{3} & \frac{1}{3} & 0 & 0 & 0 & 0\\ \frac{1}{3} & 0 & \frac{1}{3} & \frac{1}{3} & 0 & 0 & 0 & 0\\ \frac{1}{3} & \frac{1}{3} & 0 & \frac{1}{3} & 0 & 0 & 0 & 0\\ \frac{1}{3} & \frac{1}{3} & \frac{1}{3} & 0 & 0 & 0 & 0 & 0 \end{array}\right]$$
