# Supplementary material for: Graph Based, Adaptive, Multiarm, Multiple Endpoint, Two‐Stage Designs
Source: Stat Med. 2025 Dec 2;44(28-30):e70237. doi: 10.1002/sim.70237 (PMC12670376; doi:10.1002/sim.70237)
Supplement: Supplementary file 1 — Data S1. Supporting Information. [file SIM-44-0-s001.pdf]

Supplemental Appendix: Illustrations of Consonant Adaptive Tests Becoming Non-Consonant and Non-Consonant Tests Becoming Consonant

We illustrate with a simple example that the combination test method is not consonant, if no hypotheses are dropped but can become consonant, if only a single hypothesis is selected for the second stage. For the same setting we demonstrate that, in contrast, for the CER method the test is consonant if no hypotheses are dropped but may become non-consonant otherwise.

For simplicity we consider an example with a two-armed trial with two endpoints tested with a Bonferroni-Holm test, corresponding to the graph in Figure 1 and assume the correlations are unknown. In addition, we assume that an interim analysis is performed at information fraction $t = 0.5$ but that no α is spent at the interim analysis. Similar examples can be constructed in cases where the correlation is known and for trials with early rejections.

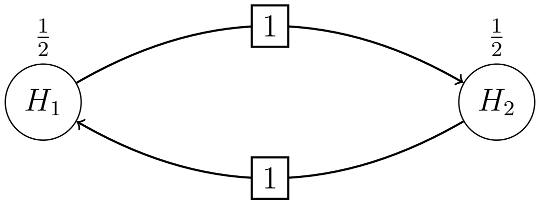

Figure 1: Graph corresponding to a Bonferroni-Holm test.

0.1 Combination Test Method

We consider two adaptation scenarios. In the first, the trial continues as pre-planned. In the second one hypothesis is dropped.

Scenario 1: The trial continues as pre-planned In this case the test can be come non consonant if in the frist stage $p_{1,1} < p_{2,1}$ but in the second stage $p_{1,(2)} > p_{2,(2)}$. Asssume, for example, that the first stage elementary p-values are $p_{1,1} = 0.05, p_{2,1} = 0.4$ and the incremental second stage p-values are $p_{1,(2)} = 0.5, p_{2,(2)} = 0.03$. Then the stagewise p-values for the intersection hypothesis $H_{\{1,2\}}$ are
$p_{\{1,2\},1} = 2\min(0.05, 0.4) = 0.1, , p_{\{1,2\},(2)} = 2\min(0.5, 0.03) = 0.06$. Then using the inverse normal combination function, the overall p-value for the intersection hypothesis is 0.0225 such that the intersection hypothesis can be rejected. On the other hand, the elementary p-values for hypotheses H_1, H_2 are 0.122 and 0.0656 and none of the elementary hypothesis can be rejected.

Scenario 2: The trial continues with the hypotheses with the smallest first stage p-value We show that for this adaptation rule that continues only with the hypotheses corresponding to the smalles first stage p-value, the combination test is consonant: Let

$p_{1,1}, p_{2,1}$ denote the first stage p-value and without limiting generality assume that $p_{1,1} < p_{2,1}$. According to the selection rule, for the second stage, the hypothesis H_2, corresponding to the larger p-value, is dropped and H_1, the hypothesis corresponding to the smaller p-value is selected.

The first stage p-value for the intersection hypothesis $H_{\{1,2\}}$ is $p_{\{1,2\},1} = \min[1, 2\min(p_{1,1}, p_{2,1})$ and the second stage p-value is $p_{\{1,2\},(2)} = p_{2,(2)}$ such that the combination test rejects the intersection hypothesis if

$$C_{\{1,2\},1}(p_{\{1,2\}}, p_{1,(2)}) \leq \alpha$$

Now, since $p_{\{1,2\},1} \geq p_{1,1}$ and the combination function is monotone, we have

$$\mathcal{C}_{\{1,2\}}(p_{\{1,2\},1}, p_{1,(2)}) \geq \mathcal{C}_{\{1,2\}}(p_{1,1}, p_{1,(2)}),$$

where the term on the right side is the combination test for the elementary test of H_1. Therefore, rejection of the intersection hypothesis in this case implies rejection of the elementary hypothesis.

0.2 Conditional Error Rate Method

As there is no early stopping, the preplanned test is a fixed sample test, defined by the second stage boundaries. For the intersection hypothesis $H_1 \cap H_2$ these are the Bonferroni adjusted boundaries $\{0.0125, 0.0125\}$ and for the elementary hypotheses H_1, H_2 the unadjusted boundary 0.025 for both tests.

As above, let $p_{1,1}, p_{2,1}$ denote the first stage p-values. We consider again two adaptation scenarios. In the first, the trial continues as pre-planned. In the second one hypothesis is dropped.

Scenario 1: The trial continues as pre-planned Let $p_{1,(2)}, p_{2,(2)}$ denote the second stage p-values and $p_{1,2}, p_{2,2}$ the corresponding cumulative p-values as defined in (3.26) of the main paper. As no adaptations have been performed, the pre-planned boundaries can be applied. Because the critical values for the intersection hypothesis test $\{0.0125, 0.0125\}$ are smaller than the critical value 0.025 for the individual tests it follows that rejection of the intersection hypothesis implies rejection of at least one elementary hypothesis in the closed test. Therefore the test is consonant.

Scenario 2: The trial continues with hypothesis 1 only We give a numeric example showing that the test may become non consonant. Assume the first stage p-values are $p_{1,1} = 0.031, p_{2,1} = 0.032$ and assume that hypothesis 2 is dropped. Then $B_1 = 0.183$ but for the intersection $B_{\{1,2\}} = 0.190$. The reason that $B_{\{1,2\}}$ is larger than B_1 is that also the relatively small p-value of Hypothesis 2 contributes to $B_{\{1,2\}}$, which is defined as the sum of the conditional error rates of the Bonferroni adjusted individual tests of H_1 and H_2.

The corresponding bounds for the cumulative p-value are 0.025 for the elementary hypothesis H_1 and 0.0268 for the intersection hypotheses $H_1 \cap H_2$.
